# Deficiency of the pattern-recognition receptor CD14 protects against joint pathology and functional decline in a murine model of osteoarthritis

**DOI:** 10.1371/journal.pone.0206217

**Published:** 2018-11-28

**Authors:** Nisha Sambamurthy, Cheng Zhou, Vu Nguyen, Ryan Smalley, Kurt D. Hankenson, George R. Dodge, Carla R. Scanzello

**Affiliations:** 1 Translational Musculoskeletal Research Center, Corporal Michael J. Crescenz Department of Veterans Affairs Medical Center, Philadelphia, Pennsylvania, United States of America; 2 University of Pennsylvania Perelman School of Medicine, Division of Rheumatology, Philadelphia, Pennsylvania, United States of America; 3 University of Pennsylvania Perelman School of Medicine, Department of Orthopedic Surgery, Philadelphia, Pennsylvania, United States of America; 4 Department of Orthopaedic Surgery, University of Michigan, Ann Arbor, Michigan, United States of America; University of Umeå, SWEDEN

## Abstract

**Objective:**

CD14 is a monocyte/macrophage pattern-recognition receptor that modulates innate inflammatory signaling. Soluble CD14 levels in knee OA synovial fluids are associated with symptoms and progression of disease. Here we investigate the role of this receptor in development of OA using a murine joint injury model of disease.

**Methods:**

10-week-old Male C57BL/6 (WT) and CD14-deficient (CD14^-/-^) mice underwent destabilization of the medial meniscus (DMM) surgery to induce OA. Joint histopathology was used to examine cartilage damage, and microCT to evaluate subchondral bone (SCB) remodeling at 6 and 19 weeks after surgery. Synovial and fat pad expression of macrophage markers (F4/80, CD11c, CD68, iNOS, CCR7, CD163 and CD206) was assessed by flow cytometry and droplet digital (dd)PCR. Changes in locomotive activity indicative of joint pain were evaluated longitudinally up to 16 weeks by automated behavioral analysis.

**Results:**

Early cartilage damage scores 6 weeks post-DMM were similar in both strains (Mean score ±SEM WT: 4.667±1.38, CD14^-/-^: 4.6±0.6), but at 19 weeks were less severe in CD14^-/-^ (6.0±0.46) than in WT mice (13.44±2.5, p = 0.0002). CD14^-/-^ mice were protected from both age-related and post-surgical changes in SCB mineral density and trabecular thickness. In addition, CD14^-/-^ mice were protected from decreases in climbing activity (p = 0.015 vs. WT, 8 weeks) observed after DMM. Changes in synovial/fat pad expression of CCR7, a marker of M1 macrophages, were slightly reduced post-DMM in the absence of CD14, while expression of CD68 (pan-macrophage marker) and CD163 (M2 marker) were unchanged.

**Conclusion:**

CD14 plays an important role in progression of structural and functional features of OA in the DMM model, and may provide a new target for therapeutic development.

## Introduction

Osteoarthritis (OA) is a leading cause of joint pain and dysfunction, affecting general mobility, functional independence and quality of life for millions. Current treatments may mask symptoms temporarily but do not target the underlying pathology of disease. This disease is characterized by pathologic changes in multiple joint tissues, including cartilage, subchondral bone, and synovium. A role for innate inflammatory pathways has been implicated, as evidenced by multiple chemokines, cytokines and other inflammatory factors in synovial fluid (SF) [1]. Of these factors, soluble CD14 (sCD14) is a macrophage-related protein detected in high levels in SF that was positively associated with severity of joint space narrowing as well as knee-pain [2]. Moreover, this molecule can sensitize fibroblast like synoviocytes (FLS) *in vitro* to inflammatory stimuli (toll-like receptor (TLR) ligands) [3]. Together these findings suggest a mechanistic role for CD14 in joint inflammation, pathology and indicate sCD14 may serve as a biomarker of disease severity.

CD14 is a glycosylphosphatidylinositol (GPI)-anchored receptor lacking a cytoplasmic tail, expressed mainly on monocytes and macrophages [4]. It acts as a pattern recognition receptor (PRR), functioning as a co-receptor for several Toll-like receptors (TLRs) [5]. CD14 binds to lipopolysaccharide (LPS)–LPS binding protein (LBP) complexes and helps activate TLR4-myeloid differentiation protein-2 (MD2) signaling, triggering the innate immune system into action [6]. TLR mediated activity has recently been implicated in stimulating murine nociceptors involved in pain generation in response to cartilage damage [7]. Furthermore, CD14 has been described to recognize and clear apoptotic cells [8]. Activated monocytes and macrophages may release a soluble form of CD14 from the cell surface by proteolytic cleavage [9], which may enhance or dampen macrophage responses [10]. The soluble form retains biological activity as a PRR, and can act on nearby cells such as endothelial cells and fibroblasts to modulate inflammatory activity [3, 11]. However, whether CD14 plays a mechanistic role in OA development or progression is unclear. Given its role in inflammation, we tested the hypothesis that disease progression would be slowed in CD14 deficient (CD14^-/-^) mice when subjected to destabilization of medial meniscus (DMM).

## Materials and methods

### Mouse strains and destabilization of medial meniscus (DMM) surgery

Mice deficient in CD14 (B6.129S4-Cd14^tm1Frm/J^ or CD14^-/-^) and C57BL/6 congenic controls (wildtype, or WT) were obtained through Jackson laboratory. The mice were group-housed (4–5 animals/cage) in standard mouse cages held in a ventilated rack. Each cage was lined with woodchip bedding, animals were supplied with food and water ad libitum, and mice were provided with paper-mash nestlets. The room was kept on a 12:12 light:dark cycle. At 10 weeks of age [12], male mice of both strains were randomly assigned to DMM or sham groups, anesthetized (ketamine 80–100 mg/kg, xylazine 4–6 mg/kg, and acepromazine 1–2 mg/kg), and then subjected to medial arthrotomy of the right knee, and the medial meniscotibial ligament (MMTL) was exposed and severed creating instability within the joint [13]. Sham operated controls underwent medial arthrotomy of the right knee without severing the MMTL. Post-surgery, animals were treated once with buprenorphine 0.1mg/kg, and then monitored daily for the first week and 3 times per week through the course of the study for signs of physical distress. If signs of distress occurred, animals were treated with buprenorphine 0.1mg/kg SC every 8 hours as symptoms persisted. This occurred only rarely in the first week, and treatment was never required for more than 24 hours. Additional age-matched naïve (completely un-manipulated) animals from both strains were used as controls. Groups of mice (n = 5–10) were euthanized by CO_2_ inhalation at time 0 (pre-operatively, 10 weeks of age), 6 weeks and 19 weeks post-surgery for microCT (n = 4–6 at each timepoint) and histopathologic analysis (all mice) as described below. These two endpoints were chosen based on previous studies which have described phasic changes in histopathology over time [13, 14]. We chose the 6 week time point to study the early development phase of OA, when mild structural and functional features of disease become apparent in most mice, and the 19 week time point to evaluate progression to advanced structural degeneration.

### Micro-CT assessment of cortical and subchondral (SCB)

For assessment of SCB, dissected knees (n = 4–6) were scanned using VivaCt-40 micro-CT scanner (Scanco-Medical, Brüttisellen, Switzerland). The settings used to scan were: voxel size = 10.5 μm, energy = 55kV, intensity = 145 μA, and integration time = 300 ms. The medial epiphysis of the proximal tibia was used as the region of interest to assess the SCB structure. The 3-dimensional reconstructed region was manually contoured to include calcified cartilage and extend to the growth plate line. To avoid osteophytes, ten percent of the medial and lateral width of the tibial plateau was excluded. Contouring was repeated every tenth slice and splined together automatically to create a volume of interest (VOI). Percent bone volume over total volume (%BV/TV), bone mineral density (BMD, mg HA/cm^3^) and trabecular thickness (Tb.Th., mm) of the SCB were assessed using the manufacturer’s (ScanCo, Inc.) software.

For assessment of cortical bone features, the right knees (n = 5) collected at time 0 were scanned using a μCT-50 scanner (Scanco-Medical, Brüttisellen, Switzerland). Scans were obtained using the following settings: voxel size = 4.4 μm, energy = 55kV, intensity = 145 μA, integration time = 1s. The mid-femur point was determined in the scout-view by measuring the length of the femur and scanning the 450 slices above and 450 slices below the midpoint of the femur. 10 slices above and below this midpoint were contoured manually to include the cortical bone and exclude the marrow space. Percent bone volume over total volume (%BV/TV), bone mineral density (BMD, mg HA/cm^3^) and polar moment of inertia (pMOI, mm^4^) of the femoral midshaft were then assessed.

### Histopathologic evaluation of structural features of OA

After CT scanning, all knees from the 6 and 19 week timepoints were decalcified and embedded in paraffin. Sections were made in the frontal plane and a mid-point section stained with toluidine blue. The stained sections were then evaluated using the modified Osteoarthritis Research Society International (OARSI) score as previously described [15, 16], by a board-certified veterinary pathologist. Using this system, the maximum possible score per cartilage surface = 15 (medial or lateral tibial plateau or femoral condyle) and the maximum total joint score = 60. Osteophyte size was measured on the medial tibia and femur using an ocular micrometer and the largest osteophyte measured was reported in micrometers (μm).

### Immunostaining of CD14 in murine knee joints

Additional formalin-fixed, paraffin embedded thin sections of knees obtained from wildtype mice were utilized to characterize CD14 expression patterns in the joint. Routine immunoperoxidase staining technique was utilized, and antigen retrieval was performed with 0.05% Trypsin at 37°C for 30 mins. Primary antibody was rabbit anti-murine CD14 (Abcam, ab106285), and isotype-matched rabbit IgG (Abcam, ab172730) was used as a control in place of the primary antibody to confirm staining specificity. After staining, sections were counterstained with 1% methyl green.

### Assessment of spontaneous behavior in mice

The mice assigned to the 19 week endpoint were subjected to longitudinal analysis of spontaneous activity prior to sacrifice. Activities such as climbing, locomotion, eating, drinking, resting and grooming were analyzed in both strains of mice (n = 4–9 mice per group) using specially designed vibration sensing platforms (laboratory animal behavior observation registration and analysis system, LABORAS (Metris, Hoofdorp, The Netherlands) [17]. Murine activity was measured at baseline (before surgical intervention), and every 4 weeks up to 16 weeks post-surgery. Activities were assessed in batches of 2 mice each day (1 mouse per platform in a specialized LABORAS cage with bedding material) in 16-hour overnight sessions. Activity was most variable during the first 2 hours as the mice acclimatized to their new environment; hence the first 2 hours of data were excluded from the analysis.

### Assessment of synovial macrophages by cytometry and gene expression

For flow cytometric analysis of cell populations, additional C57BL/6 mice were subjected to DMM surgery, and anterior fat pad and synovium (combined) was dissected post-mortem at 4 and 8 weeks post-DMM, and collected from both the DMM operated and contralateral un-operated limbs. Tissues were pooled from 4–5 mice per sample, and cells isolated by Liberase digestion (Sigma-Aldrich, 1 Unit/ml) for 2 hrs. After isolation, cells were stained with the Live/Dead Fixable Violet Dead Cell Stain Kit (Invitrogen) and the following antibodies: CD45-PerCP Cy5.5, CD11c-Super Bright 645, F4/80-APC, iNOS-Alexa Fluor 488, CD206-PE (obtained from Thermofisher). Multicolor flow cytometry was performed on an LSR II flow cytometer (BD) with BD FACSDiva software and data analyzed with FlowJo software (Version 10). Gating strategy was performed as follows: After gating on live cells/singlets, the CD45^+^ cell population was identified, and then F4/80^+^ and CD11c^+^ cell populations were gated on CD45^+^ cells. Finally, iNOS^+^ and CD206^+^ cells were identified on F4/80^+^ and CD11c^+^ populations.

Additional mice of both strains were required for gene expression analysis of macrophage markers. Anterior fat pad and synovium was harvested at 1, 2, 4, 8 and 16 weeks post-DMM, as well as from naïve mice at baseline (10–12 week old), and tissues were pooled from 4–5 mice per sample to obtain adequate RNA. cDNA was synthesized from mRNA by routine methods, and transcript copy number quantified using the QX200 Droplet Digital PCR System (BioRad, Hercules, CA). Primers specific for CD14, CD11c, CD68, F4/80, CCR7, iNOS, CD163 and CD206 were utilized for amplification, and expressed relative to TATA-box binding protein (TBP) transcripts. 6 (pooled) samples per group were prepared and analyzed, and the operated side (right) compared to the un-operated side (left).

### Statistical analysis

Murine joint histology, activity and microCT data from three treatment groups (naïve, sham and DMM) within a strain were analyzed by the non-parametric Kruskal Wallis test, followed by Dunn’s multiple comparisons test comparing specific groups. Differences in these outcomes between two groups (i.e. between strains, same treatment) were evaluated using the Mann-Whitney U test. Longitudinal activity data were evaluated using two-way ANOVA analysis. Cell proportions and gene expression levels were compared between DMM-operated and contralateral (un-operated) limbs using Wilcoxon’s matched-pair summed rank test. P-values (corrected for multiple comparisons when appropriate) ≤ 0.05 were interpreted as statistically significant.

### Ethics statement

Animals were housed at the University of Pennsylvania and the Corp. Michael J. Crescenz VA Medical Center (CMCVAMC), and all experiments were approved by the Institutional Animal Care and Use Committees (IACUC) at both institutions (University protocol # 805383, CMCVAMC protocol #01462). Experiments were carried out in accordance with the Guide for Care and Use of Laboratory Animals of the National Institutes of Health. Animals were euthanized by carbon-dioxide over exposure following the American Veterinary Medical Association (AVMA) guidelines for euthanasia.

## Results

### Adverse events

There were five adverse events within one week of surgery. Two mice (one of each strain) died immediately from complications of anesthesia, and three CD14^-/-^ mice died from wounds inflicted by fighting with cage mates. All five had been allocated to the sham group, and were removed from the study. One naïve CD14^-/-^ mouse was euthanized and removed from the study due to a rectal prolapse.

### CD14 expression in wild type mice

**Fig 1:**
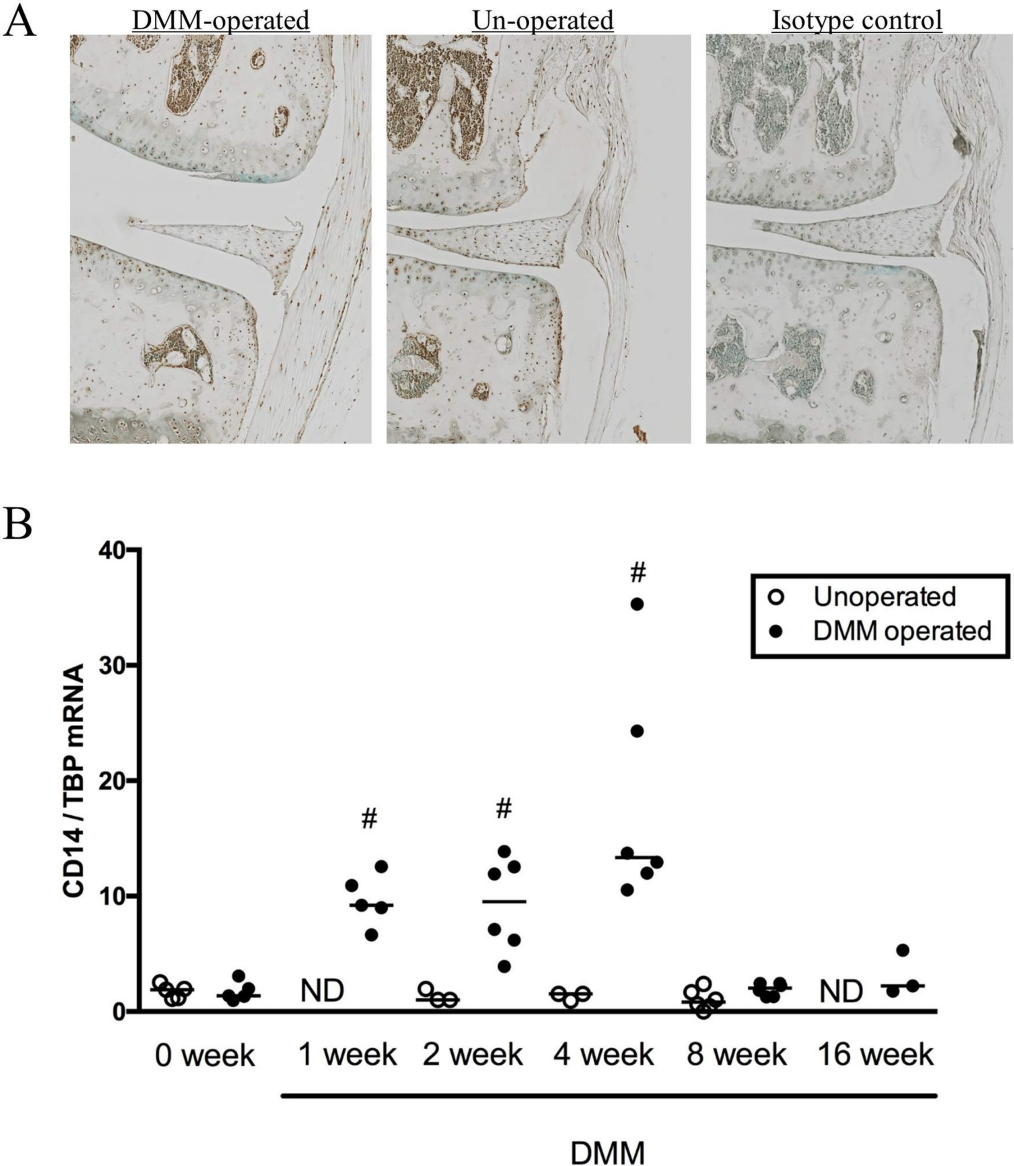
CD14 expression in wild type mice. **A.** Representative immunostaining for CD14 in a DMM-operated and un-operated joint 8 weeks post-surgery demonstrates staining in multiple cells and tissues including chondrocytes of the articular cartilage, meniscus, and the synovium/joint capsule. Medial tibial-femoral joint compartment is shown (20X). **B.** Synovial CD14 mRNA expression pre- and post-DMM surgery. CD14 transcript levels were measured in DMM operated and un-operated contralateral knees pre-operatively (0 week) and up to 16 weeks post-DMM in WT mice. mRNA copy number is reported relative to TBP transcript copy number. Line indicates median, n = 3–6 pooled tissue specimens/group, each specimen containing tissues from 4–5 individual mice. # p ≤0.05 compared to 0 week levels, Kruskal-Wallis multiple comparison test followed by Dunn’s post-test.

To determine the distribution of CD14 expression in the mouse joint, immunostaining for CD14 was performed on knee sections from WT mice subjected to DMM-surgery. As seen in **Fig 1A**, expression was widespread throughout the joint and observed on chondrocytes, meniscal cells, and in synovium and joint capsular tissues. Immunostaining did not reveal clear changes in expression patterns on the DMM-operated or contralateral un-operated limbs (8 weeks post-surgery in **Fig 1**). mRNA levels were then measured in synovial/fat pad tissues harvested post-mortem from WT mice pre-DMM and up to 16 weeks post-DMM, and levels compared to the un-operated (left) limbs (**Fig 1B**). mRNA expression increased compared to baseline only on the DMM-operated side, and peaked at 4 weeks post-DMM returning to baseline levels by 8 weeks.

### Cartilage changes and osteophytosis post-DMM in wild type and CD14 deficient mice

**Fig 2:**
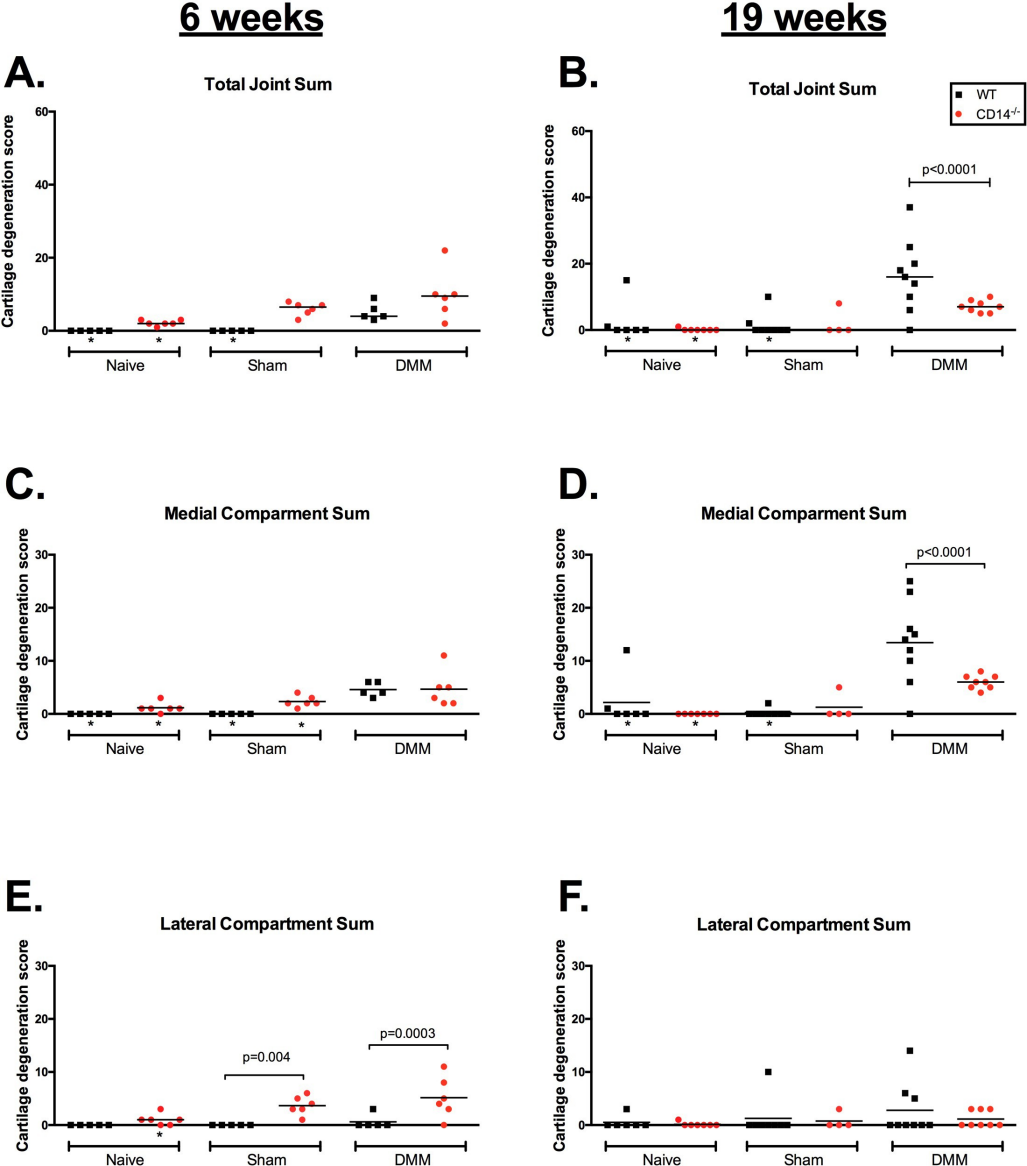
Cartilage degeneration in WT and CD14^-/-^ mice at 6 weeks (early phase) and 19 weeks (advanced phase) post-DMM surgery. Cartilage degeneration was scored as described. Wild type mice are in black, CD14^-/-^ in red. Total joint cartilage degeneration sum at 6 weeks **(A)** and 19 weeks **(B).** Medial compartment (femoral and tibial) sum, at 6 weeks **(C)** and 19 weeks **(D)**. Lateral compartment (femoral and tibial) scores at 6 weeks **(E**) and 19 weeks **(F)**. P-values indicate statistically significant differences between strains. * Multiplicity adjusted p<0.05 compared to DMM-operated group of same strain. N = 5–10 mice/group.

**Fig 3:**
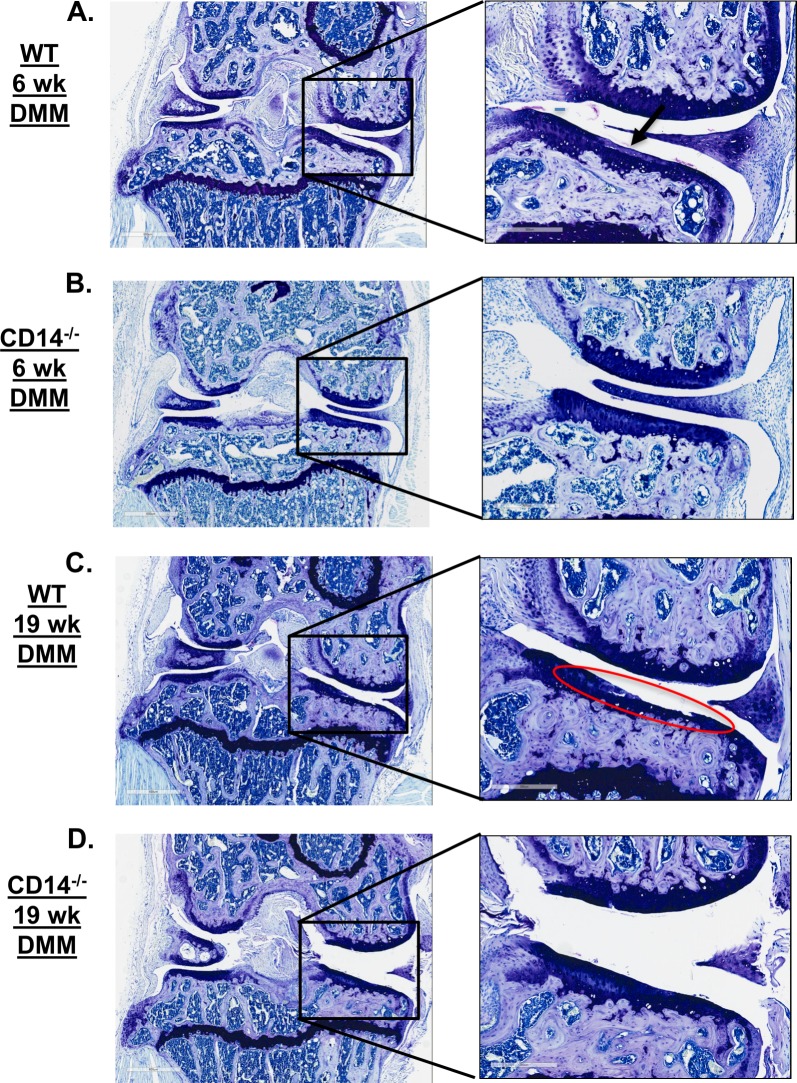
Knee histopathology of WT and CD14 deficient mice post-DMM. Representative photomicrographs show histopathology of the whole knee joint (left) and medial compartment (right) in (**A**) WT mouse at 6 weeks post-DMM, (**B**) CD14^-/-^ mouse at 6 weeks post-DMM, (**C**) WT mouse at 19 weeks post-DMM and (**D**) CD14^-/-^ mouse at 19 weeks post-DMM.

Knee cartilage histopathology was evaluated using a well-established semi-quantitative scoring system [15, 16] at 6 and 19 weeks post-surgery to evaluate the contribution of CD14 on early development and later progressive phases of disease. 6 weeks post-surgery, WT mice showed mild cartilage damage (Mean total joint cartilage score ±SEM: 5.2±2.39) as compared to sham-operated (0±0, p = 0.008) or un-operated controls (0±0, p = 0.008) (**Fig 2A)**. At this early stage, CD14^-/-^ mice subjected to DMM surgery had slightly greater (although not statistically significantly) cartilage damage compared with WT mice (CD14^-/-^: 9.83±2.74, p>0.99). Some sham-operated CD14^-/-^ mice also showed slight cartilage damage (Mean score CD14^-/-^ sham: 6±0.73 p>0.99 compared to CD14-/- DMM, **Fig 2A**). Separate analysis of the medial and lateral cartilage surfaces (tibial and femoral combined, max score = 30) demonstrated that cartilage damage in CD14 deficient mice at this early stage was observed in both medial and lateral compartments, while it was mostly constrained to the medial compartment in the wild type strain (**Fig 2C and 2E**). As anticipated in this model, 19-weeks post-DMM cartilage damage in WT mice was significantly greater (16.22±3.61, p = 0.02) compared to the 6-week time point (**Fig 2B**), and still largely seen in the medial compartment (**Fig 2D and 2F**). In contrast, cartilage damage in DMM-operated CD14^-/-^ mice was not significantly different at 19 weeks (7.125±0.64) compared to six weeks, and was significantly lower than the WT strain at the same time point (16.22±3.6,1p<0.0001) (**Fig 2B**). Representative photomicrographs of knee joints at 6 weeks (**Fig 3A and 3B**) and 19 weeks (**Fig 3C and 3D**) after DMM surgery are presented in **Fig 3**.

**Fig 4:**
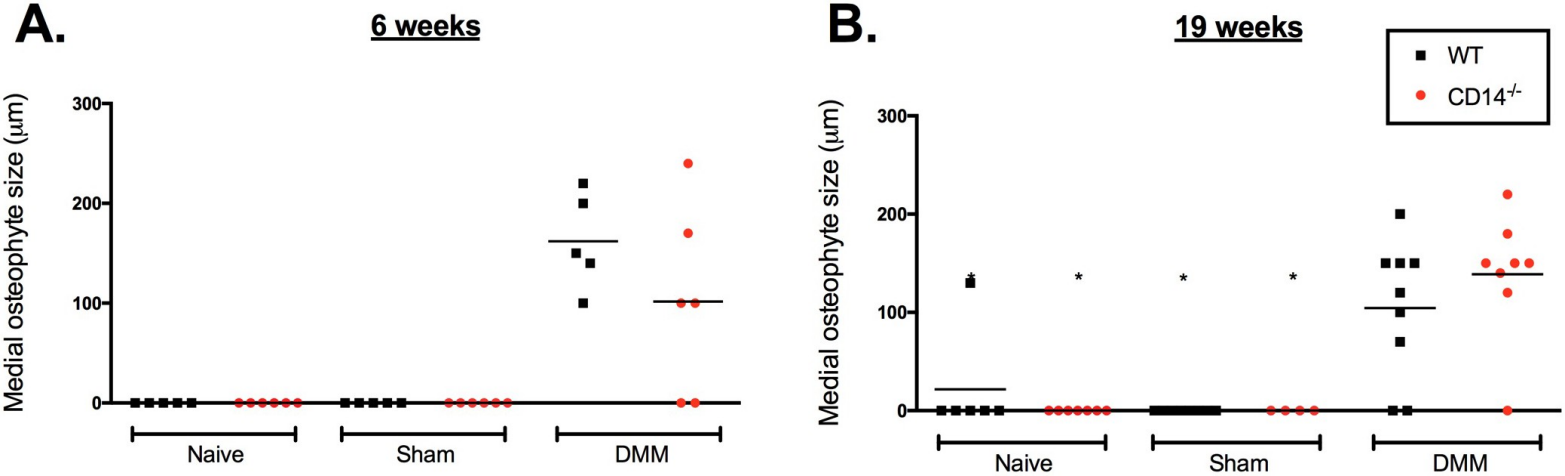
Osteophyte size in CD14 deficient and wildtype mice after DMM. Osteophyte size measured using an ocular micrometer (as described in Materials and Methods) at 6 weeks (**A**) and 19 weeks (**B**) post-DMM. * Multiplicity adjusted p-value≤0.05, calculated by Kruskal-Wallis test followed by Dunn’s post-test comparing individual groups. n = 5–10 mice/group.

Osteophytes were measured on the medial side of the knee joint. Osteophyte size was significantly larger in the DMM group compared to naïve or sham-operated controls within each strain at both time points. Mean size of osteophytes in CD14^-/-^ mice at both 6 weeks (101.7±38.51mm) and 19 weeks post-DMM (138.8±22.48 mm) was not statistically different than WT mice (6 weeks: 162±21.54 mm, p = 0.64 c/w CD14^-/-^, 19 weeks: 104.4±23.16 mm, p>0.99) (**Fig 4**). Overall, osteophyte size was variable in both strains post-DMM, and was not significantly between 6 and 19 weeks in either strain.

### Cortical and subchondral bone differences between WT and CD14 deficient mice

**Fig 5:**
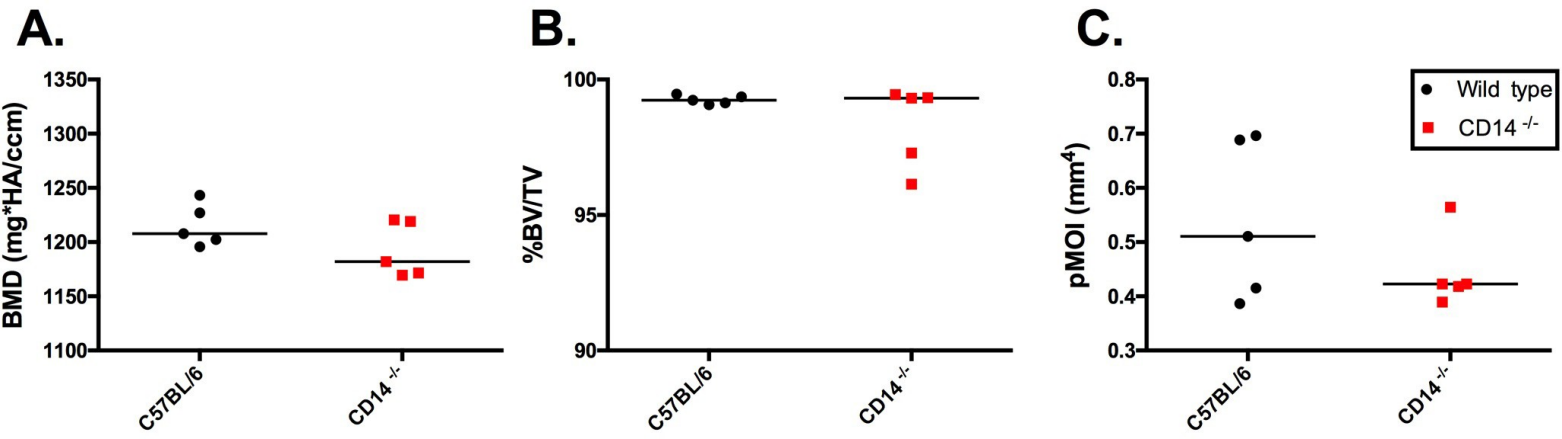
Micro-CT analysis of cortical bone in 10 week old WT and CD14^-/-^ mice. Panels **A, B and C** display bone mineral density (BMD), percent bone volume to total volume (%BV/TV) and polar moment of inertia (pMOI) respectively. Values are expressed as median, n = 5 per group. No statistically significant differences between strains were observed (Mann-Whitney U test).

**Fig 6:**
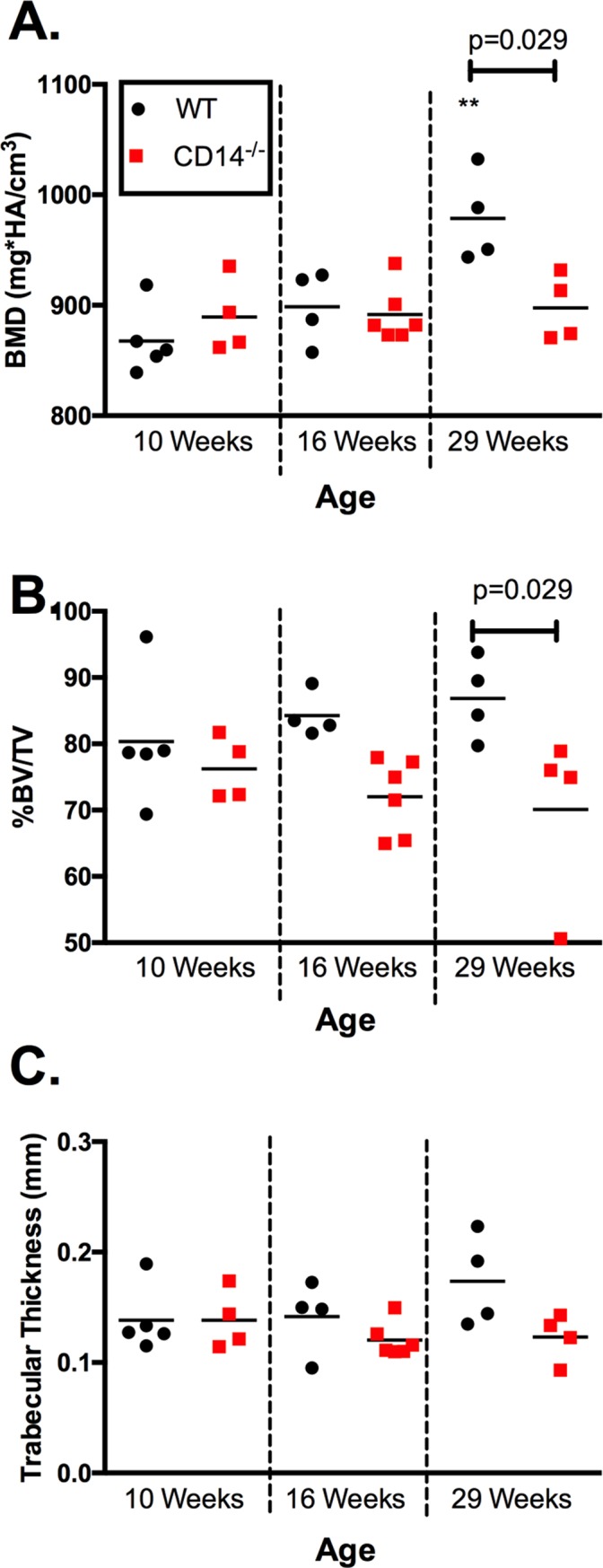
Subchondral bone outcomes in naïve mice. Micro-CT scans of naïve joints at 10 weeks (baseline), 16 weeks (6 weeks post in the model) and 29 weeks of age (19 weeks post) were performed in both strains. n = 4–6 mice per group. WT mice in black circles, and CD14^-/-^ mice are in red squares. ** Indicates p<0.05 compared to 10 weeks of age in same strain, Kruskal-Wallis multiple comparison test followed by Dunn’s post-test.

**Fig 7:**
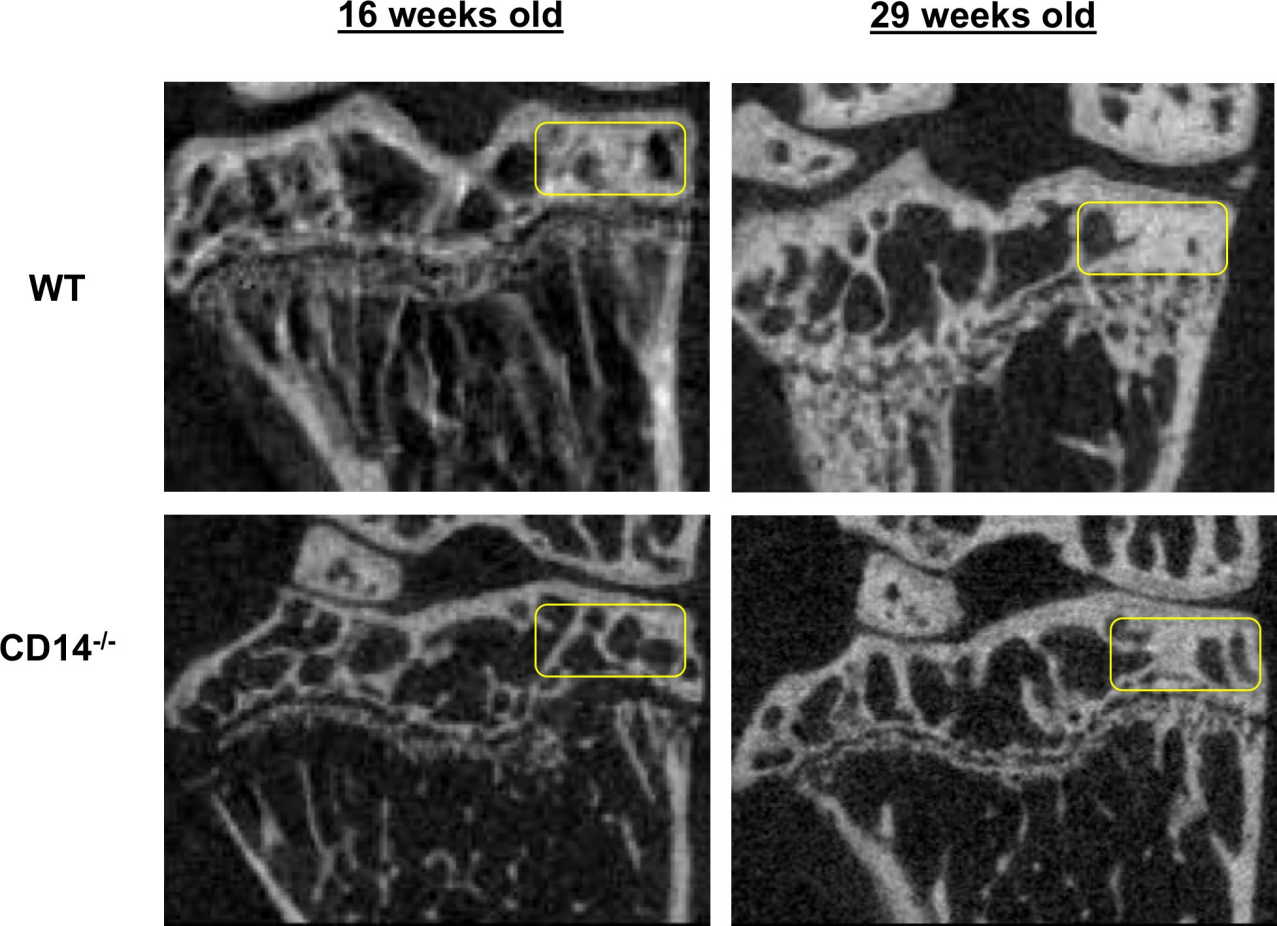
MicroCT images of subchondral bone in naïve WT and CD14 deficient mice. Representative microCT scans of unoperated naïve WT (top) and CD14^-/-^ mice (bottom) at 16 and 29 weeks of age. Yellow boxes indicate the subchondral region analyzed for the outcomes presented in **Fig 5**(BMD, %BV/TV, Tb.Th).

MicroCT analysis of femoral cortical bone (BMD, %BV/TV and pMOI) demonstrated comparable cortical bone quality in naïve mice of both strains at 10-weeks of age (**Fig 5**). We then evaluated the medial tibial subchondral plate, as it is a major site of bone remodeling in OA both in humans and in the DMM model [18, 19]. Micro-CT analysis of naïve joints at baseline (10 weeks of age) and 16 weeks of age (time = 6 weeks in the model) revealed no significant differences in SCB properties (BMD, %BV/TV and trabecular thickness) between strains (**Fig 6**), similar to cortical bone findings. However, by age 29 weeks (time = 19 weeks in the model) WT naïve male mice showed age-related increases in BMD (978.7±20.42 mg HA/cm^3^) compared to baseline (867.7±13.49, p = 0.006), while no increases with age were observed in the CD14^-/-^ strain. BMD in WT mice was significantly higher than in CD14 deficient mice at 29 weeks old (p = 0.029, **Fig 6A**). Percent bone volume fraction (%BV/TV) showed a slight but non-significant age-related increase in naïve wild-type mice, and was significantly greater at 29 weeks of age in comparison to CD14-deficient mice (p = 0.029) (**Fig 6B**). No significant age- or strain-related differences in trabecular thickness (Tb.Th.) were observed (**Fig 6C**). Representative 2-dimensional microCT images from each strain at 16 and 29 weeks of age are shown in **Fig 7**, with the area analyzed indicated.

### Surgery-related subchondral bone changes in the two strains

**Fig 8:**
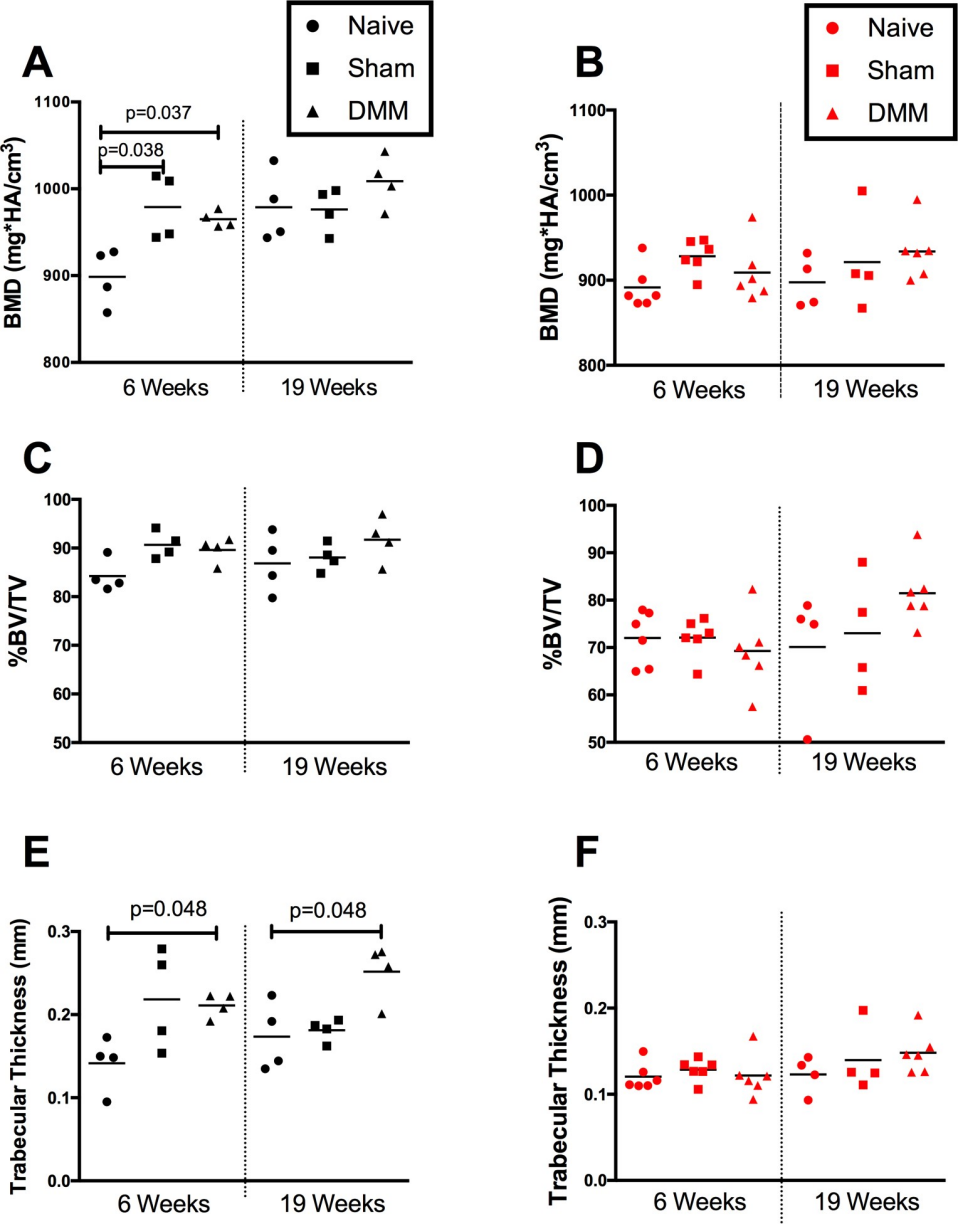
Micro-CT analysis of SCB in naïve and operated (sham or DMM) joints at 6 and 19 weeks post-surgery. Panels **A**, **C and E** depict trends in BMD, %BV/TV and Tb.Th respectively in WT mice. Panels **B**, **D and F** indicate trends in CD14^-/-^ mice. (n = 4–6 mice per group). P-values (multiplicity adjusted) obtained by Kruskal-Wallis multiple comparison test followed by Dunn’s post-test.

Six weeks post-DMM, BMD increased in operated WT mice compared to naïve controls (DMM: 946.9±4.7, naïve: 898.7±16.45, p = 0.037) (**Fig 8A**), while %BV/TV was maintained (**Fig 8C**). The sham operated WT group also displayed increased BMD (979.0±19.02, p = 0.038 compared to naïve). Similar increases in Tb.Th. were observed post-DMM in WT mice compared to naïve controls (p = 0.048) at both time points (**Fig 8E**). Interestingly, the CD14-deficient mice maintained baseline levels of BMD (**Fig 8B**), % BV/TV (**Fig 8D**), as well as Tb.Th. (**Fig 8F**) after DMM surgery at both 6 and 19 weeks.

### Macrophage populations in WT mice post-DMM

**Fig 9:**
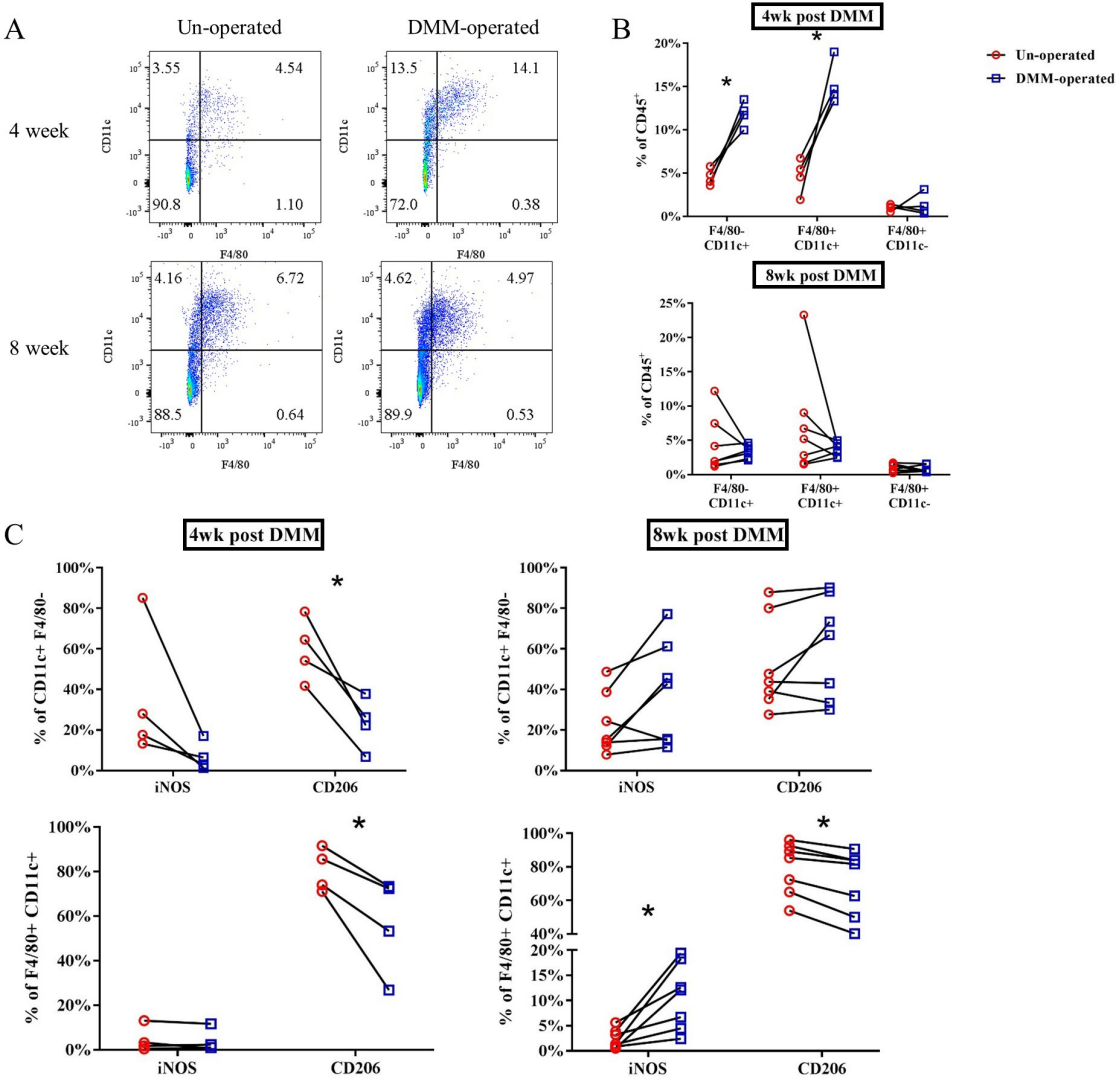
Flow cytometric analysis of synovial/fat pad tissues from mice. **A**: Representative CD11c and F4/80 expression gated on the CD45^+^ cell population from DMM- & un-operated SM/FP tissue at 4- & 8-week post-DMM. **B**: Quantification of F4/80^+^ & CD11c^+^ cell populations as % of CD45+ gate. **C**: iNOS^+^ & CD206^+^ cell populations expressed as percent of CD11c^+^ F4/80^-^ (top panels) & CD11c^+^ F4/80^+^ cells (bottom panels) at 4- & 8-week post-DMM. (n = 4 for 4-week post-DMM, n = 7 for 8-week post-DMM. Each specimen contained cells pooled from 4–5 individual mice. *p<0.05 compared to un-operated side, Wilcoxon matched-pairs summed rank test.)

Synovium and infrapatellar fat pad are commonly infiltrated by macrophages in OA. To confirm whether myeloid-lineage cells such as macrophages are increased in joint tissues after DMM surgery, we performed flow cytometric analysis on cells isolated from dissected synovial/fat pad tissues from WT mice 4 and 8 weeks after DM surgery, as described in the Methods. Cells were first gated on CD45 expression (hematopoietic lineage marker) and expression of the myeloid markers F4/80 (pan-macrophage marker) and CD11c (expression on macrophages and dendritic cells) examined [20]. As shown in **Fig 9A**, two main populations of CD45^+^ cells were defined by F4/80 and CD11c expression: A CD11c^+^ F4/80^-^ population (likely enriched with dendritic cells based on previous reports [21], and a CD11c^+^ F4/80^+^ population (likely enriched with macrophages [22]. Percentages of both populations were significantly increased in DMM-operated compared to un-operated joints at 4 weeks but not at 8 weeks (Fig 9A and 9B, F4/80^-^ CD11c^+^, DMM: 11.84±1.46%, un-operated: 4.55±0.98, p = 0.009%. F4/80^+^ CD11c^+^, DMM: 15.28 ± 2.55%, un-operated: 4.66±2.06%, p = 0.017). Expression of iNOS and CD206 expression were next evaluated as markers indicative of M1 and M2 macrophage phenotypes respectively [23] on the CD11c single positive, and CD11c^+^ F4/80^+^ populations. Compared to the un-operated side, CD206^+^ F4/80^+^ CD11c^+^ cells and CD206^+^ F4/80^-^ CD11c^+^ cells were significantly lower proportionally in DMM-operated limbs at 4 weeks, and this trend was sustained at 8 weeks in the F4/80^+^ CD11c^+^ population (**Fig 9C**). Percentage of iNOS^+^ cells were slightly elevated in the F4/80^+^ CD11c^+^ macrophage population at 8 weeks (p = 0.02) post-DMM, but overall numbers of cells were small.

### Macrophage gene expression in WT mice and comparison with flow cytometry

**Fig 10:**
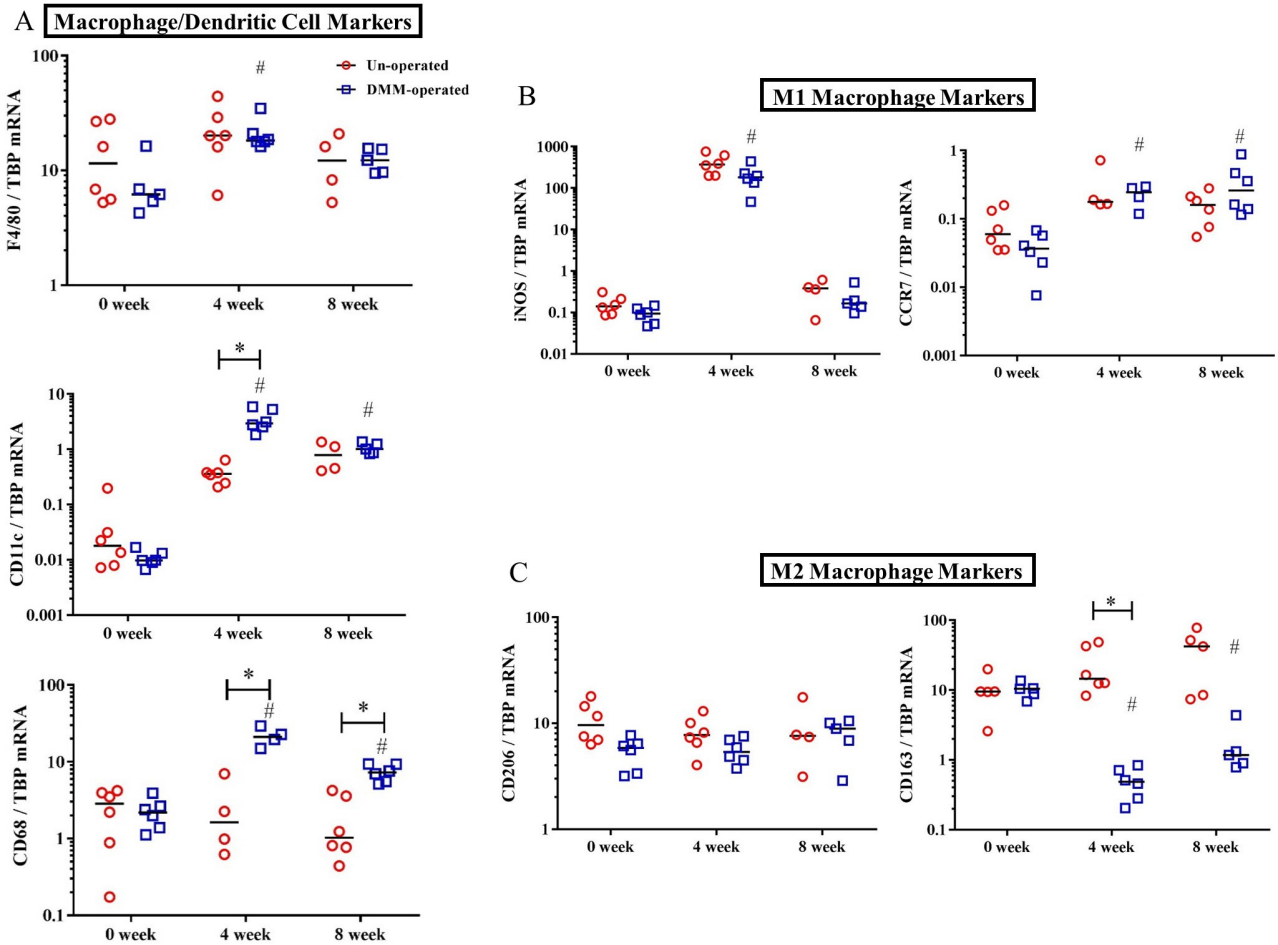
Macrophage-related gene expression in WT mice at 4 and 8 weeks post-DMM. Transcript levels of F4/80, CD11c, and CD68 (pan-macrophage/dendritic cell markers) (**panel A**), iNOS and CCR7 (M1 macrophage markers, **panel B**), CD206 and CD163 (M2 macrophage markers, **panel C**) in dissected synovial/fat pad tissues from operated and contralateral un-operated knees were measured at baseline (0 week), 4 week and 8 weeks post-DMM in WT mice. mRNA copy number measured by droplet digital PCR is expressed as a ratio to TBP transcripts. Line indicates median, n = 4–6 specimens/group, each specimen containing tissues from 4–5 individual mice. *p<0.05 compared to un-operated side, Wilcoxon matched-pairs summed rank test. # p ≤0.05 (multiplicity adjusted) compared to baseline of same strain, Kruskal-Wallis multiple comparison test followed by Dunn’s post-test.

To determine whether similar phenotypic changes post-DMM could be detected at the mRNA level, we measured multiple markers of macrophage lineage (F4/80, CD68, and CD11c) and phenotype (M1: iNOS, CCR7; M2: CD206, CD163) [21–25] by qPCR. CD11c and CD68 expression levels were increased on the DMM-operated side at 4 weeks post-DMM compared to the un-operated side (CD11c: 9.8-fold higher; CD68: 9.7-fold higher than un-operated, all p<0.05), and CD68 remained elevated (3.3-fold higher, p<0.05) at 8 weeks post-DMM while F4/80 expression levels did not differ at either time point (**Fig 10A**). All three markers were elevated compared to baseline levels at 4 weeks, and CD68 and CD11c remained elevated at 8 weeks. Of the M1-markers, mRNA levels for iNOS did not differ significantly between DMM- and un-operated sides at 4 or 8 weeks, while levels of CCR7 mRNA were slightly elevated on the DMM-operated side at 8 weeks (0.35±0.12, p = 0.01, **Fig 10B**). However, levels of iNOS were highly upregulated compared to baseline at 4 weeks (2000-fold higher, p<0.05, **Fig 10B**). Levels of CCR7 mRNA were much lower overall than iNOS, but CCR7 mRNA was upregulated compared to baseline at both 4 (6.6-fold) and 8 weeks (7-fold). Transcripts for the M2 marker CD163 were highly expressed at baseline, and substantially lower on the DMM-operated side at 4 weeks (30-fold lower) and 8 weeks (35- fold lower than un-operated, both p<0.05, **Fig 10C**). No changes in levels of CD206 mRNA were observed. Overall there were similarities and differences between cytometric and gene expression analysis. Specifically, F4/80 and CD206 transcripts were not highly modulated despite clear modulation in proportions of cells expressing these markers on the DMM-operated side, while iNOS mRNA expression was very highly expressed at 4 weeks post-DMM, despite minimal expression on F4/80^+^ and CD11c^+^ cells by cytometry. Of the macrophage markers, CD68 and CD11c mRNA expression patterns more closely reflected measured changes in F4/80 and CD11c expressing cell populations in this model, while CCR7 and CD163 mRNA levels more closely paralleled post-DMM changes in M1-type (iNOS^+^ cells) and M2-type populations (CD206^+^ cells).

### Comparison of macrophage gene expression in WT and CD14 deficient mice post-DMM

**Fig 11:**
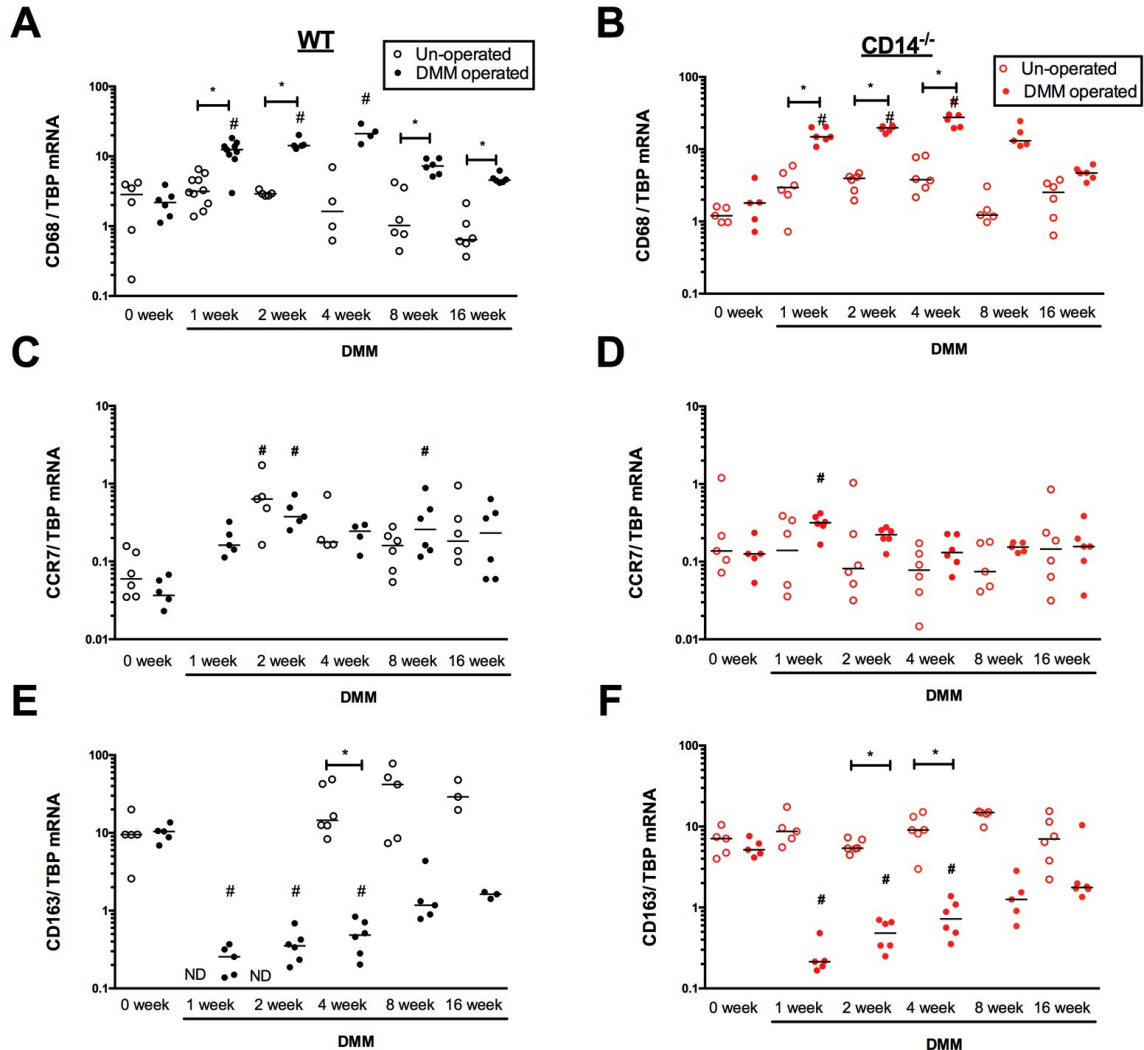
Macrophage transcript levels in synovial/fat pad tissues from WT and CD14^-/-^ mice. CD68 (pan-macrophage marker), CCR7 (pro-inflammatory macrophage marker) and CD163 (anti-inflammatory macrophage marker) transcript levels in dissected synovial/fat pad tissues from operated and contralateral un-operated knees were measured at baseline (0 week) and up to 16 weeks post-DMM in WT (**panels A, C and E**) and CD14^-/-^ mice (**panels B, D and F**). mRNA copy number was measured by droplet digital PCR and expressed as a ratio to TBP transcripts. Line indicates median, n = 4–10 specimens/group, each specimen containing tissues from 4–5 individual mice. *p<0.05 compared to un-operated side, Wilcoxon matched-pairs summed rank test. # p ≤0.05 (multiplicity adjusted) compared to baseline of same strain, Kruskal-Wallis multiple comparison test followed by Dunn’s post-test.

We compared CD68 mRNA levels (**Fig 11A**) as a pan-macrophage marker between WT and CD14^-/-^ strains out to 16 weeks post-DMM. Levels were increased in DMM-operated limbs of WT mice compared to preoperative levels with peak expression at 4 weeks post-surgery (21.61±3.02). After 4 weeks, levels remained significantly elevated compared to the un-operated limb up to 16 weeks post-DMM (DMM: 4.77±0.31, contralateral: 0.90±0.26). CD68 expression levels and temporal patterns were similar in the CD14^-/-^ strain (**Fig 11B**). Next, CCR7 mRNA levels were measured as a marker of the M1 phenotype [20]. Overall CCR7 expression levels were much lower and highly variable compared to CD68. CCR7 expression increased after DMM surgery (**Fig 11C**), and was significantly elevated in operated limbs compared to pre-operative (0.04±0.01) levels at 2 weeks (0.44±0.08, p = 0.0006) and 8 weeks (0.35±0.12, p = 0.01). Expression remained elevated in a subset of mice up to 16 weeks post-DMM, so that mean CCR7 transcript levels were still 6-fold higher (0.27±0.10 p = 0.06, **Fig 11C)** than baseline although this difference was not statistically significant. Interestingly, increases in CCR7 transcripts post-DMM were also observed on the un-operated side, but were statistically significant compared to baseline only at 2 weeks post-surgery (p = 0.003, **Fig 11C)**. In CD14^-/-^ mice, baseline CCR7 levels were higher compared to WT (WT: 0.04±0.01, CD14^-/-^: 0.13±0.03, p = 0.02). Significant elevations post-DMM were only observed on the operated side at 1-week post-DMM in CD14^-/-^ mice (0.31±0.04) and were not sustained at later time points (**Fig 11D**). Finally, we analyzed expression of CD163, a macrophage scavenger receptor expressed by anti-inflammatory (M2) macrophages [21]. In WT mice, statistically significant decreases in expression compared to pre-operative levels (10.06±0.01) were observed after DMM surgery at 1 week (0.25±0.05, p = 0.0003), 2 weeks (0.37±0.07, p = 0.002) and 4 weeks (0.5±0.1, p = 0.013), with levels starting to approach baseline thereafter. Expression on the un-operated side was comparable to baseline (**Fig 11E**) at all post-operative times examined (4, 8 and 16 weeks). CD14^-/-^ mice displayed similar trends in CD163 expression as their WT counterparts (**Fig 11F**), with CD163 expression being reduced at baseline (CD14^-/-^: 5.56±0.62) compared to the WT strain (10.06±0.11, p = 0.016).

### Spontaneous activity patterns at baseline and post-surgery in CD14^-/-^ and WT mice

**Fig 12:**
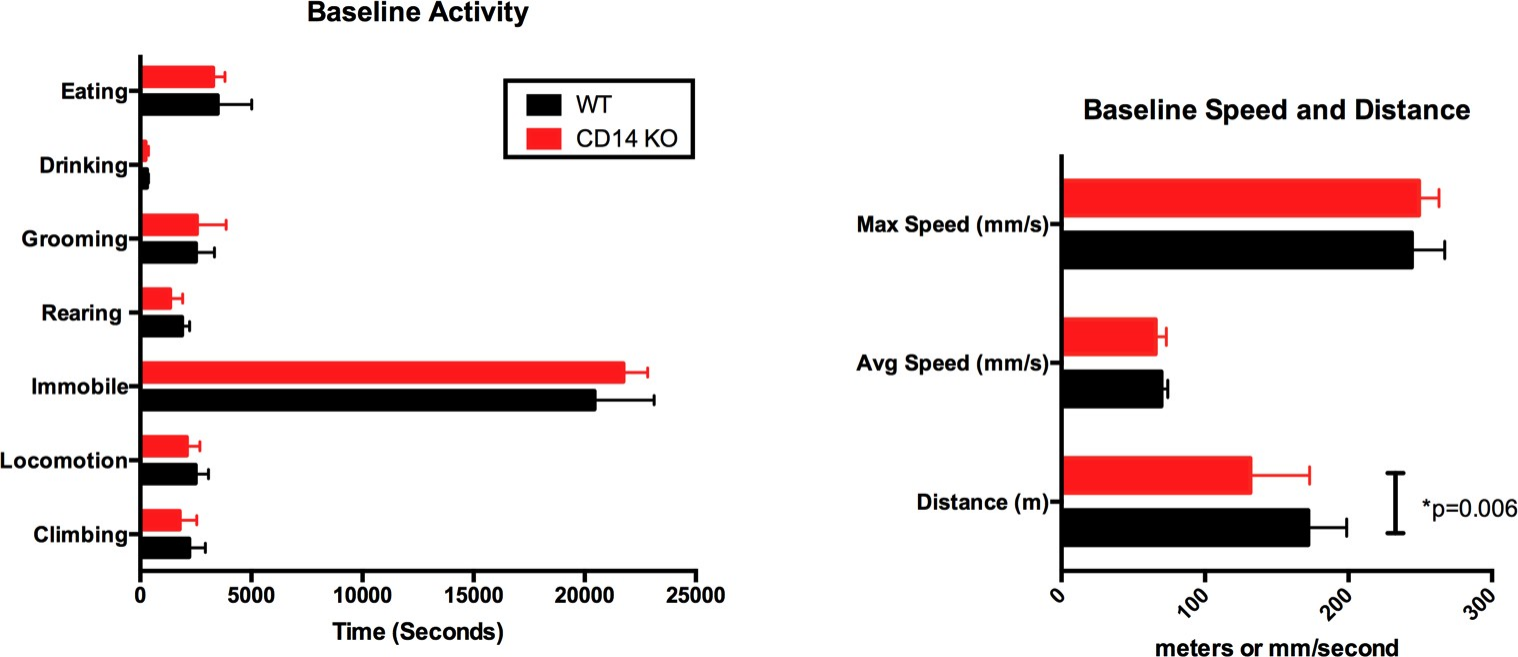
Baseline activity and speed in wildtype and CD14 deficient strains. **(A)** Time spent in various activities or immobile were measured using the LABORAS system as described in Materials and Methods. 10 week old mice from both strains were compared. No significant differences between strains were observed. **(B)** Maximum and average speed during locomotion, and total distance traveled in 14 hours, were calculated. CD14^-/-^ mice covered less distance than WT mice.

Reduction in spontaneous activities indicative of movement-induced pain has been reported in WT mice after DMM surgery [26, 27]. We measured multiple spontaneous murine activities to evaluate if the absence of CD14 expression altered pain-related behavior post-DMM [17]. At Baseline, activities were measured in 23 WT and 23 CD14^-/-^ mice a week before surgery. Quantification of time spent eating, drinking, grooming, climbing, rearing and in locomotion, indicated no differences between strains (**Fig 12A**). There was also no significant difference in speed of travel during locomotion (**Fig 12B**), although CD14^-/-^ mice covered less distance in 14 hours (135.85±8.16 m) than their WT counterparts (170.66±8.97 m, p = 0.006, **Fig 12B**).

### Longitudinal assessment of pain-related behaviors post-DMM

**Fig 13:**
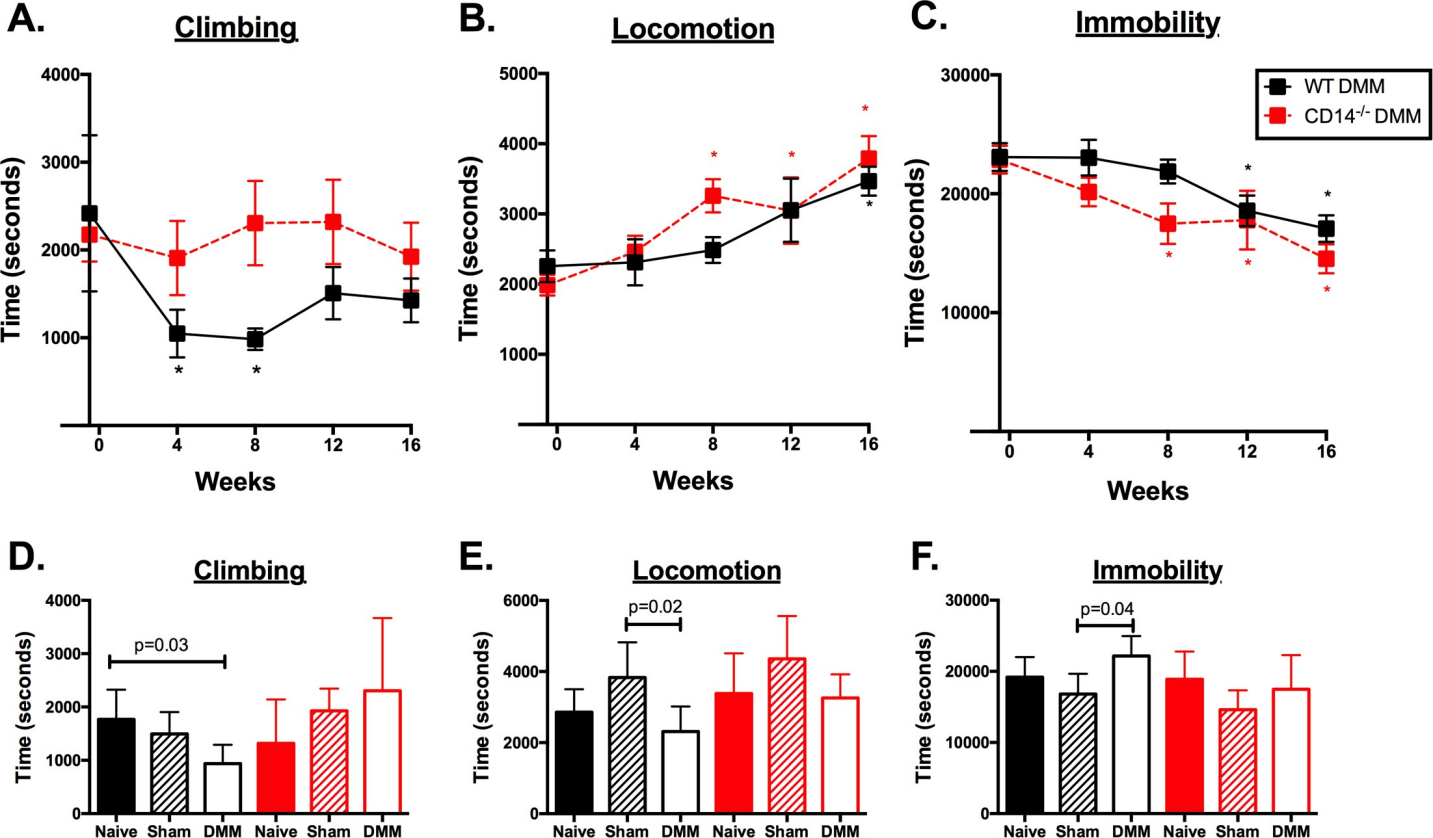
Spontaneous activities in WT and CD14 deficient mice. Longitudinal trends in climbing (**A**), locomotion (**B**) and immobility (**C**) were measured pre-operatively and every 4 weeks post-DMM up to 16 weeks. * p≤0.05, 1-way ANOVA with Dunn’s post-test comparing baseline (pre-operative) activity levels within each strain. Maximal differences were observed in climbing activity, where WT mice (black square) showed significant decreases in activity by 4 and 8 weeks post-DMM while CD14^-/-^ mice (red squares) maintained climbing activity throughout the time course. **(D-F):** Spontaneous activities at 8 weeks post-DMM compared to control groups in both strains. Significant decreases in climbing (**D**) and locomotion (**E**), and increases in immobility (**F**) were observed 8 weeks post-DMM in WT mice compared to controls. P-values obtained by Kruskal-Wallis followed by Dunn’s post-test. n = 4–9 mice/group. CD14^-/-^ mice showed no significant differences compared to controls in these outcomes post-DMM at 8 weeks). P-values obtained by one-way ANOVA followed by Dunn’s post-test. n = 4–9 mice/group. Results presented as mean±SEM.

The two strains showed the most distinct differences in climbing activity longitudinally. While WT mice showed significant decreases compared to baseline in climbing by 4 and 8 weeks post-DMM, the CD14^-/-^ mice maintained climbing activity over the course of 16 weeks (**Fig 13A**). Both strains displayed increases in locomotion over time, although these increases occurred as early as 8 weeks post-DMM in CD14^-/-^ mice, while increases in locomotion compared to baseline were delayed in WT mice until 16 weeks post-DMM (**Fig 13B**). Time being immobile showed the opposite trend in both strains, with decreases post-DMM. These decreases occurred earlier (at 8 weeks) in CD14^-/-^ mice compared to WT mice which were not significantly decreased from baseline until 12 weeks post-DMM. (**Fig 13C**).

### Activities post-DMM compared to controls in both strains

The most significant difference in activity levels between strains were observed at 8 weeks post-surgery. DMM-operated WT showed significantly decreased time spent climbing at 8 weeks post-surgery (939.3±117.5 secs) compared to un-operated naive controls (1768±196.3 secs, p = 0.03) (**Fig 13D**), as well as reduced time (2315±235 secs) in locomotion compared to sham-operated controls (3832±404.2 secs, p = 0.02, **Fig 13E**). This decreased activity was reflected in greater immobility 8 weeks post-DMM (22158±932.8 secs) in WT mice compared to controls (sham: 16821±1163 secs, p = 0.04). In contrast, CD14 deficient mice displayed no significant reductions in climbing (**Fig 13D**) or locomotion (**Fig 13E**) or increases in immobility 8 weeks post-DMM (**Fig 13F**) compared to their controls. No significant differences compared to controls were observed in either strain at the other timepoints (**S1 Fig**).

## Discussion

CD14 is a cell-surface receptor that facilitates inflammatory signaling via toll-like receptors (TLRs) [5]. TLR signaling during tissue damage and stress is hypothesized to be an important driver of inflammation in OA [28]. CD14 is highly expressed on the surface of monocytes [29], and can be cleaved from the surface of cell to become soluble. The soluble product (sCD14) retains biological activity and can interact with TLRs expressed on surrounding cells [8, 11]. Previously, we demonstrated relevance of this pathway to sustained inflammatory activity in the OA joint, as high levels of sCD14 in OA synovial fluids potentiated TLR inflammatory signaling and cytokine production from synovial fibroblasts *in vitro* [3]. Others have shown that levels of sCD14 and sCD163 in synovial fluids are independently associated with both pain severity and radiographic progression of disease in OA patients [2, 30]. The current study expands on these observations by demonstrating a functional role for CD14 in structural and symptomatic disease progression in an *in vivo* model.

Inflammation is implicated as a driver of catabolic activity in cartilage [31], and our current results add additional support. CD14 deficient mice show decreased inflammatory responses to lipopolysaccharide induced cardiac damage [32], and show decreased lethality in response to certain gram-negative bacterial infections [33]. In this study, we tested the effect of CD14 deficiency on OA development after injury using the DMM model. Interestingly, the effect of CD14 deficiency on cartilage damage in this model was dependent on time from surgery (**Fig 2**). Onset of articular cartilage damage evaluated at 6 weeks post-DMM surgery was equivalent between WT and CD14 deficient mice, but the subsequent progression of cartilage damage observed at 19 weeks post-DMM in WT mice did not occur in the absence of CD14. By 19 weeks average cartilage scores in CD14^-/-^ mice were 56% (2.3 fold) less severe than wild type counterparts. These results implicate CD14 as important in progression, but not initial establishment, of cartilage lesions post-injury and highlight the importance of evaluating multiple time points in pre-clinical models. The DMM model used for this study is well suited for identification of stage-dependent effects, as disease progression occurs more slowly than other models [13].

Although CD14 is highly expressed by monocyte/macrophage lineage cells, expression has been documented on normal human chondrocytes [34], and in this study we demonstrate expression in murine chondrocytes (**Fig 1**). Future studies will determine whether effects of CD14 deficiency on progression of cartilage erosion are direct or indirect. It has been suggested that targeting inflammation will have maximum effect if initiated early before onset of cartilage damage, to significantly impact the future development of OA [35]. Our current results raise the possibility that specific inflammatory pathways, perhaps downstream of CD14, may provide therapeutic targets that allow disease modification even if initiated after onset of cartilage damage. As only a subset of patients with OA present for medical attention at the earliest stages, this is an intriguing possibility.

Osteoarthritis is a complex joint disease, causing pathology in multiple joint tissues [36]. For this reason, we evaluated pathologic changes in bone, synovium and fat pad, and found that CD14 deficiency had differential effects in these tissues compared to cartilage. The formation of osteophytes, for example, was largely similar between the two strains of mice (**Fig 4)**. Work in other models suggests that cartilage erosion and osteophytosis may be driven by independent mechanisms [37]. In the collagenase model of OA, osteophytosis was dependent on synovial macrophage activity [38]. Our data demonstrates no effect on expression of CD68, and minimal differences in CCR7 and CD163, indicating similar macrophage infiltration and phenotypes in the two strains (**Fig 11**). This might explain why CD14 deficiency had minimal if any effect on osteophytosis.

Subchondral bone sclerosis is also common in OA patients and recapitulated in the DMM model. CD14 deficiency completely protected mice from post-DMM SCB changes (**Fig 8**). As opposed to findings in cartilage, SCB protection occurred early and was maintained. Of interest, WT mice showed age-related increases in BMD that were similar in magnitude to increases after DMM surgery (**Fig 6**). CD14 knockout animals did not undergo these age-related changes. These findings together point towards a role for CD14 in regulating bone mineralization and sclerosis in OA as well as aging. CD14^+^ cells are involved in both osteoclast and osteoblast activity. Human osteoclasts are derived from CD14^+^ monocytes [39], and CD14 plays a functional role in LPS-stimulated bone resorption [40]. But stimulation of TLR4 also activates CD14^+^ monocytes to produce oncostatin M (OSM), inducing osteoblast differentiation and matrix mineralization by human mesenchymal stem cells [41]. It is possible that in the DMM model the effect on osteoblastogenesis dominates, leading to net SCB formation in response to surgery, and blunting of this response in the absence of CD14. As mechanisms behind SCB remodeling both in the model and in human OA are poorly understood, this warrants further investigation.

CD14 deficient mice did not develop activity deficits seen in the WT strain post-DMM, most obvious with climbing activity (**Fig 13**). Deficits in spontaneous activities including climbing were previously demonstrated to be related to pain in this model [26, 27]. Macrophages play a well-known role in driving inflammatory pain [42] providing a potential link between CD14 activity and pain-related functional deficits. In addition, TLRs (which interact with CD14) are expressed by nociceptors, and endogenous TLR ligands expressed in OA joints were shown *in vitro* to directly activate nociceptive neurons [7]. These CD14 knockout mice show decreased mechanical allodynia and thermal hyperalgesia after spinal nerve L5 transection [43]. It is possible that CD14 deficiency prevents functional decline in this model via both direct effects on TLR-mediated nociception, and indirect effects of inflammation on pain. In our current study, CD14 deficiency prevented functional deficits even in the early stage of disease despite development of cartilage lesions in this strain. Although a correlation between cartilage structural and functional decline is reported, the current data suggests that structure and function can be differentially impacted by inflammatory dysregulation. These findings have important implications for translation of anti-inflammatory approaches from OA models to patients.

Given the role of CD14 in monocyte/macrophages, we evaluated macrophage-related gene expression. In OA, the synovial membrane and intra-articular fat pads in the joint become thickened and infiltrated with macrophages [31], so expression was examined in these tissues. Although previous reports suggest minimal synovial inflammation in the DMM model, our combined use of sensitive PCR methodology with confirmation of cellular distribution by flow cytometry was likely instrumental in revealing subtle signs of inflammation not easily identified otherwise. Cytometric analysis in WT mice at 4 and 8 weeks post-DMM (**Fig 9**) revealed time-dependent increases in F4/80^+^ CD11c^+^ macrophages as well as F4/80^-^ CD11c^+^ dendritic cells in SM/FP tissues after DMM surgery, peaking at 4 weeks. These changes in cell proportions were reflected by changes at the transcriptional level of select (CD68 and CD11c) but not all macrophage markers (**Fig 10**). Overall proportions of CD206^+^ macrophages were much higher than iNOS^+^ cells, indicating that an M2-type phenotype predominates in WT murine SM/FP tissues. Significant decreases compared to the un-operated side at 4 and 8 weeks post-DMM in macrophages expressing CD206 suggest early and sustained suppression of the M2 reparative phenotype in the model. Although M1-type iNOS-expressing macrophages were slightly increased at 8 weeks post-DMM, this reflected expression on very small proportions of cells. Importantly, not all phenotypic markers measured at the mRNA level reflected changes in these markers on F4/80^+^ CD11c^+^ macrophages. Specifically, CD206 transcript levels were not modulated in the model despite clear differences in proportions of cells expressing these markers, while iNOS mRNA expression was highly upregulated more than other markers despite minimal expression measured on macrophages. In this study, iNOS mRNA changes might reflect expression by other unmeasured cell populations, or post-transcriptional or translational mechanisms may be more important in controlling cellular expression of this gene on macrophages. It is quite revealing that mRNA levels of other M1 and M2 phenotypic markers, specifically CCR7 and CD163 (**Fig 10**), more closely reflected proportions of macrophages expressing other M1 and M2 markers detected by cytometry in this model. Given large numbers of mice required to extract sufficient cell numbers for cytometry, reports in OA rodent models have relied heavily on gene expression patterns to describe macrophage phenotypes [44, 45]. The discrepancies between transcriptional and cellular analysis seen in this study point to the importance of choosing macrophage phenotypic markers carefully when using gene expression as an outcome.

Our comparison of 16-week macrophage gene expression patterns between WT and CD14 deficient mice (**Fig 11**) focused on markers (CD68, CCR7 and CD163) that most closely reflected changes in macrophage populations in the model (**Fig 10**). This analysis revealed no difference in the time course or magnitude of CD68 increase in the CD14 deficient strain, suggesting that overall macrophage infiltration was not impacted by loss of this receptor.

Additional phasic alterations in synovial/fat pad gene expression post-DMM paralleled changes observed in cell populations, with upregulation of the M1 marker CCR7 and substantial downregulation of the M2 marker CD163. An increase in CCR7 expression was observed in WT mice after DMM surgery (**Fig 11**), with levels remaining somewhat elevated although variable out to 16 weeks. Interestingly, this post-DMM increase was also observed on the un-operated side suggesting a bilateral or systemic component. Pre-operative expression of CCR7 was slightly higher in the CD14 deficient strain, and elevations post-DMM were only observed at 2 weeks and not sustained. Whether these subtle differences contribute to the protection in cartilage degeneration seen later in the model remains to be determined. CCR7 may be expressed by lymphocytes and dendritic cells as well, so the specific cell types involved need further clarification. As opposed to CCR7, significant and sustained reductions in the M2 marker CD163 were observed in operated limbs after DMM, and were comparable between strains over the time-course. Taken together, results indicate that CD14 deficiency could alter the balance between M1 and M2 differentiation and activity in the joint during the post-DMM inflammatory response, without altering overall macrophage infiltration. CD14 deficient macrophages produce significantly less IL-6 and TNFα upon *in vitro* stimulation [46] in comparison to wildtype macrophages. But whether differences in macrophage cytokine production between the two strains explain outcomes needs further validation. Modern imaging techniques have shown that the burden of activated macrophages in the joint is associated with symptom severity and progression in OA [47], so a role for CD14 in macrophage activation in this model is a distinct possibility.

There are limitations to this study that deserve consideration. The use of global knockouts does not allow one to determine whether the observed effects are due to direct or indirect effects of CD14 on specific tissues within the joint. Moreover, the effect of CD14 deletion on later stage cartilage damage suggests a phasic importance of this receptor, but alterations in mechanisms driving disease during global deficiency might influence how phases progress in the model. These questions can be resolved in future work utilizing cell-specific and conditional expression models. Most macrophage lineage and phenotype markers can be expressed by other cell types such as granulocytes and dendritic cells, so expression of M1 and M2 transcripts by cells other than macrophages cannot be ruled out. Indeed, our cytometric analysis confirms that the F4/80^-^ CD11c^+^ population, which is likely enriched with dendritic cells, also expresses CD206 and iNOS [26]. Analysis of multiple myeloid cell types will therefore be an important component of future studies of inflammation in this model. Despite these limitations, this is the first study implicating CD14 activity in driving multiple pathologic features and functional outcomes in an OA model. Our observations strengthen the growing data on importance of innate inflammatory pattern-recognition receptors and mechanisms in OA, and highlight the significance of assessing both structural and functional disease outcomes at multiple time points in pre-clinical models. Recognition of the phasic effects on different joint tissues will be important in timing of interventions and evaluation of outcomes in future studies.

## Supporting information

S1 Table Histopathology data. (XLSX)

S2 Table MicroCT data. (XLSX)

S3 Table Synovial gene expression data. (XLSX)

S4 Table Longitudinal activity data. (XLSX)

S5 Table Weight data. (XLSX)

S6 Table Flow cytometry data. (XLSX)

S7 Table PCR primers table. (XLSX)

S1 Fig Climbing, locomotion and immobility compared to control groups at 4, 12 and 16 weeks post-DMM. Time spent climbing at **(A)** 4, **(B)** 12 and **(C)** 16 weeks. Time spent in locomotion at **(D)** 4, **(E)** 12 and **(F)** 16 weeks. Time spent immobile at **(G)** 4, **(H)** 12 and **(I)** 16 weeks.
(TIFF)

S1 File ARRIVE checklist. (DOCX)
