# Supplementary figures and images for: Deficiency of the pattern-recognition receptor CD14 protects against joint pathology and functional decline in a murine model of osteoarthritis
Source: PLoS One. 2018 Nov 28;13(11):e0206217. doi: 10.1371/journal.pone.0206217 (PMC6261538; doi:10.1371/journal.pone.0206217)

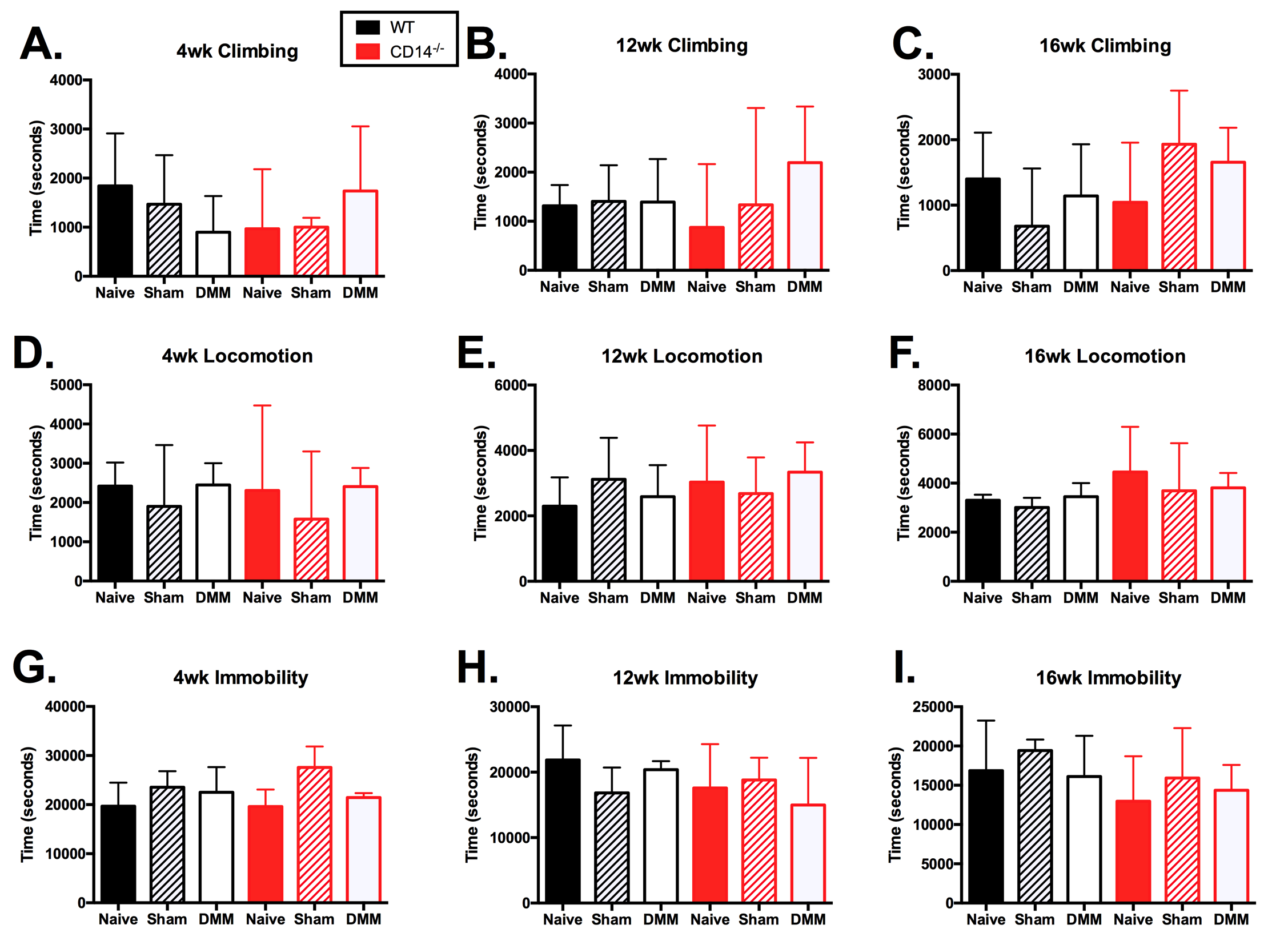

Supplement: S1 Fig — Time spent climbing at (A) 4, (B) 12 and (C) 16 weeks. Time spent in locomotion at (D) 4, (E) 12 and (F) 16 weeks. Time spent immobile at (G) 4, (H) 12 and (I) 16 weeks. (TIFF) [file pone.0206217.s008.tiff]
