# Supplementary material for: Deficiency of the pattern-recognition receptor CD14 protects against joint pathology and functional decline in a murine model of osteoarthritis
Source: PLoS One. 2018 Nov 28;13(11):e0206217. doi: 10.1371/journal.pone.0206217 (PMC6261538; doi:10.1371/journal.pone.0206217)

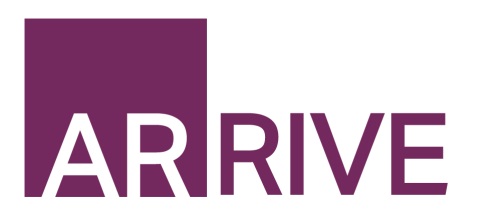


The ARRIVE Guidelines Checklist

Animal Research: Reporting In Vivo Experiments

Carol Kilkenny^1^, William J Browne^2^, Innes C Cuthill^3^, Michael Emerson^4^ and Douglas G Altman^5^

*^1^The National Centre for the Replacement, Refinement and Reduction of Animals in Research, London, UK, ^2^School of Veterinary Science, University of Bristol, Bristol, UK, ^3^School of Biological Sciences, University of Bristol, Bristol, UK, ^4^National Heart and Lung Institute, Imperial College London, UK, ^5^Centre for Statistics in Medicine, University of Oxford, Oxford, UK.*

|  | | ITEM | RECOMMENDATION | Section/ Paragraph |
| --- | --- | --- | --- | --- |
| 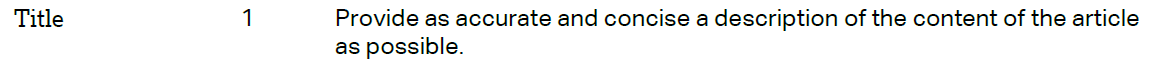 | | | Title page (1), Line 4 |  |
| 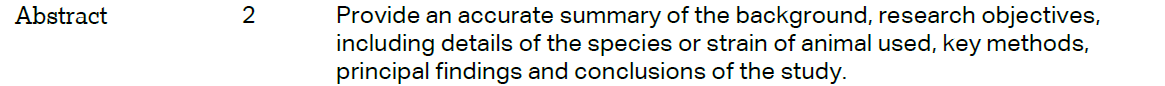 | | | Abstract page (2), Lines 45-67 |  |
| INTRODUCTION | | |  |  |
| 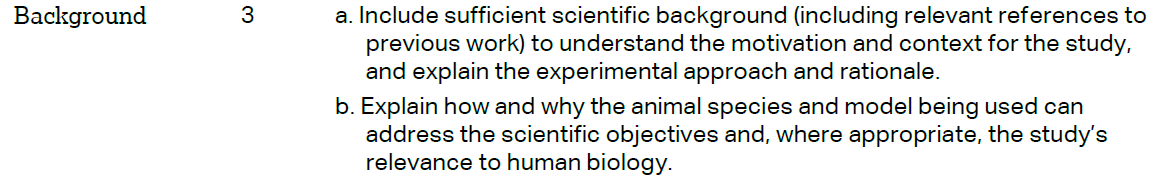 | | | Introduction, Pages 3-4, lines 69-98  Discussion, page 19, lines 448-450 |  |
| 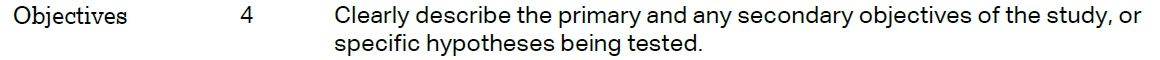 | | | Introduction, Page 4, Lines 94-98 |  |
| METHODS | | |  |  |
| 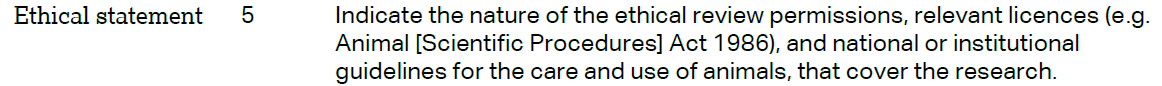 | | | Materials and Methods, Pages 8-9, Lines 200-207 |  |
| 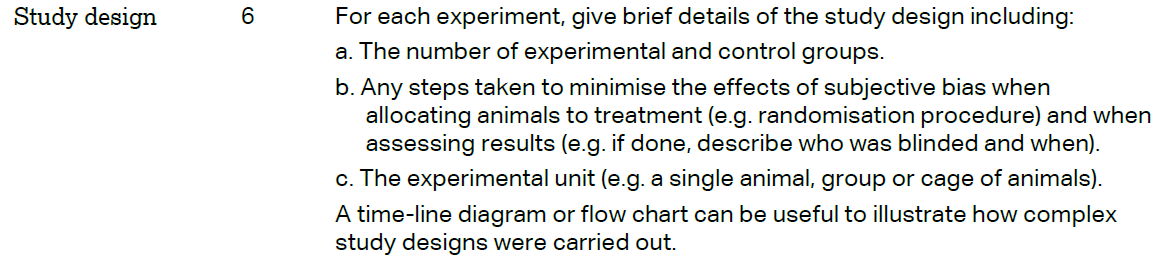 | | | Materials and Methods, Lines 119-127, 130, 163-170, 177-187  Figure legends |  |
| 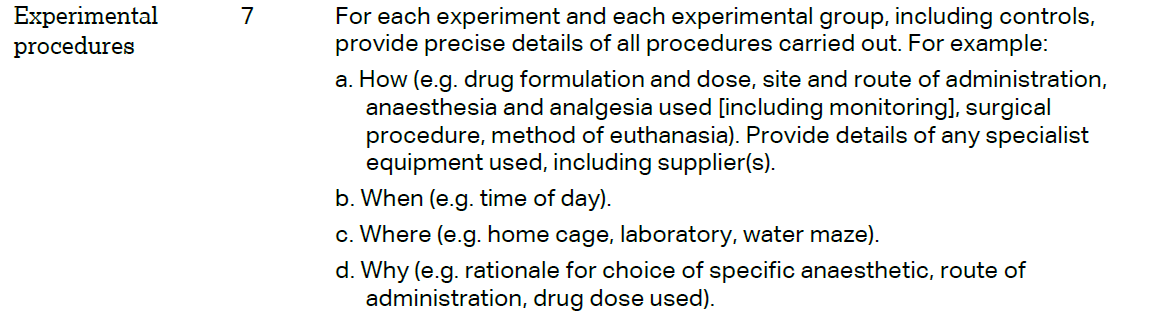 | | | Materials and Methods, Lines 102-118, 163-172 |  |
| 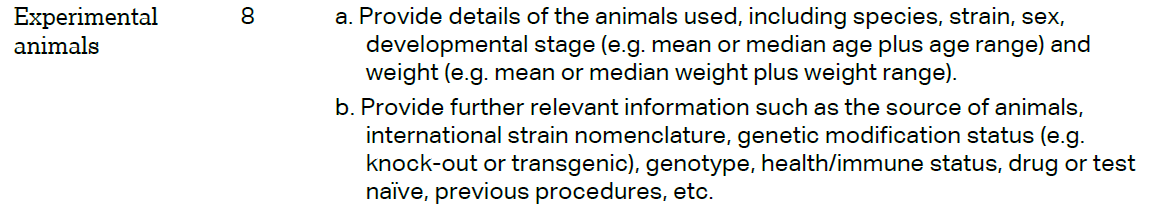 | | | Materials and Methods, Lines 102-104, 108-109 |  |

The ARRIVE guidelines. Originally published in *PLoS Biology*, June 2010^1^

| 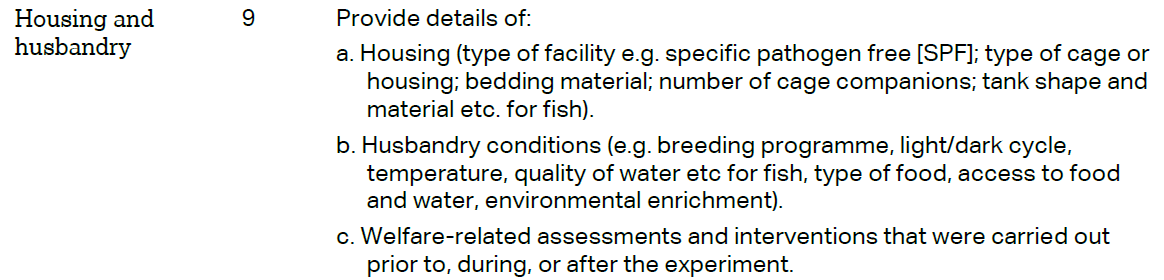 | Materials and Methods, Lines 102-117 | |
| --- | --- | --- |
| 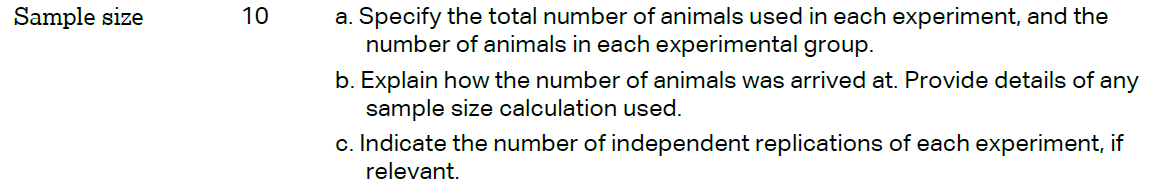 | Materials and Methods, Lines 119-122, 130, 164-168, 177-187  Figure legends | |
| 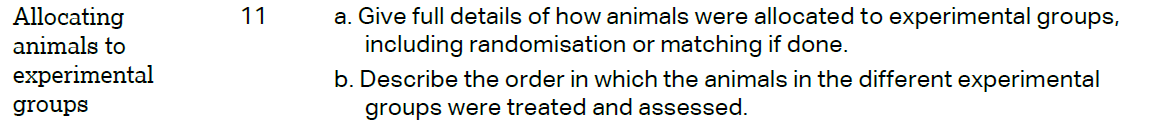 | Materials and Methods, Lines 108-127 | |
| 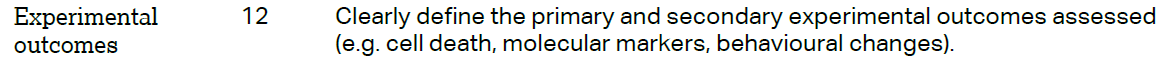 | Materials and Methods, Lines 129-187 | |
| 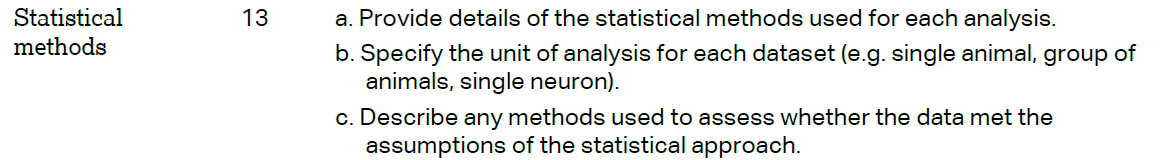 | Materials and Methods, Lines 189-198 | |
| RESULTS |  | |
| 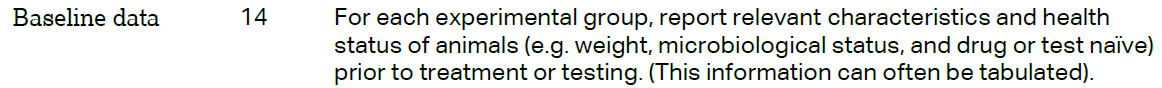 | Supplemental Table 6 | |
| 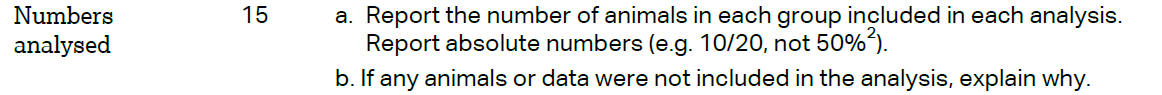 | Results, Lines 215-422 and Figure legends | |
| 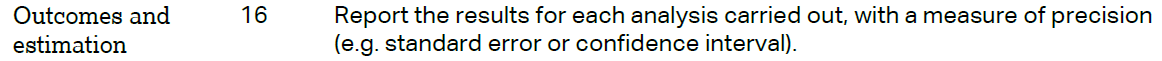 | Results, Lines 215-422, and Figure Legends | |
| 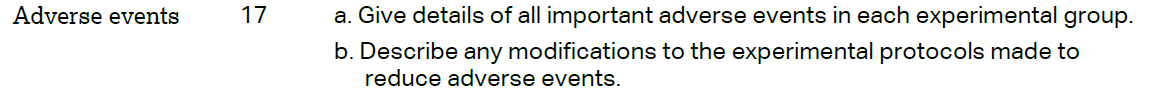 | Results, Lines 210-214 | |
| DISCUSSION |  | |
| 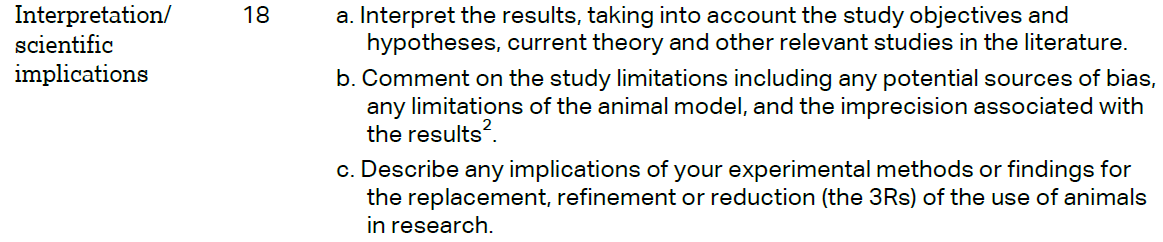 | Discussion, Lines 424-553 | |
| 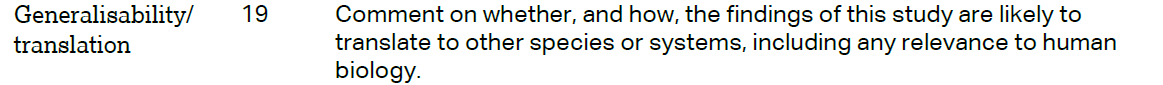 | Discussion, Lines 451-452, 459-463, 501-507 | |
| 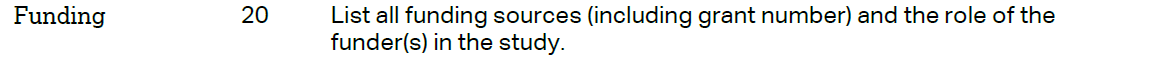 | | Title page, Lines 34-41 |


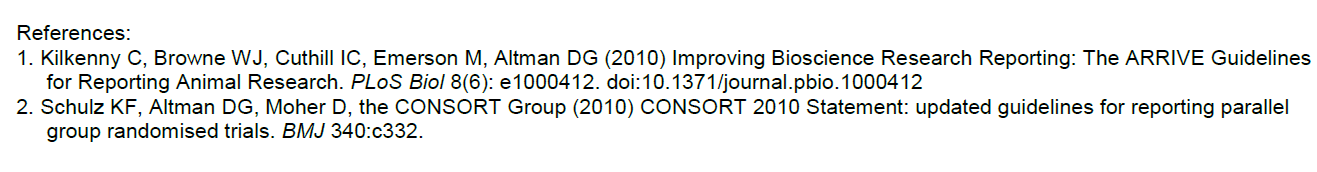

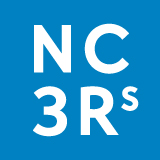

Supplement: S1 File — (DOCX) [file pone.0206217.s009.docx]
